# Accelerate Flash Removal of PFAS from Soil by Human-Guided Bayesian Optimization and Interpretable Machine Learning

**DOI:** 10.1021/acsnano.5c20063

**Published:** 2026-03-23

**Authors:** Jingbo Qin, Yi Cheng, Jayathilake Malinda, Yufeng Zhao, James M. Tour, Jian Lin

**Affiliations:** † Department of Mechanical and Aerospace Engineering, 14716 University of Missouri, Columbia, Missouri 65211, United States; ‡Department of Chemistry, §Department of Materials Science and NanoEngineering, ∥Smalley-Curl Institute, Nano Carbon Center and the Rice Advanced Materials Institute, 3990 Rice University, Houston, Texas 77005, United States; ⊥ 7038 Corban University, 5000 Deer Park Drive SE, Salem, Oregon 97317, United States

## Abstract

Flash Joule heating (FJH) presents an attractive method to decompose per- and polyfluoroalkyl substances (PFAS) but suffers from an optimization challenge due to its complex reaction dynamics. In this study, we introduce a data-driven workflow that includes a Human-Guided Bayesian Optimization (HGBO) algorithm and an interpretable multibranch neural network (MBNN) to understand and optimize PFAS removal from soil. The HGBO algorithm incorporates expert intuition into the optimization cycle via a probabilistic acquisition strategy to enhance efficiency. In two iterations, HGBO improves the PFAS removal efficiency by 60%, outperforming vanilla BO and human-centered optimization. The results are well interpreted by SHapley additive expansion (SHAP) values and partial dependence analysis (PDA) to quantify feature significance and interactions. An interpretable MBNN is then developed to quantify the contributions of functional groups in various PFAS to the FJH degradation mechanism, which is further validated by density functional theory calculations. Seamless integration of HGBO and interpretable MBNN in one data-driven workflow not only accelerates experimental optimization but also provides interpretability, enabling more informed experimental decisions in complex chemical synthesis with limited data.

## Graphical abstract

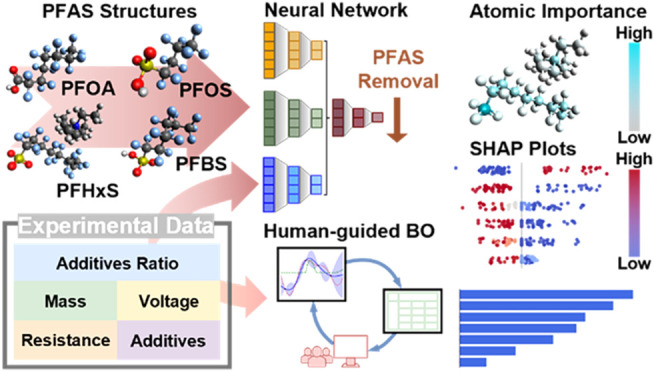

## 1 Introduction

Per- and polyfluoroalkyl substances (PFAS) are persistent environmental pollutants. Due to their high chemical stability and resistance to degradation, PFAS readily accumulate in soils, posing significant threats to ecosystems and human health through soil-to-water and soil-to-food transfer pathways.^1–3^ Effective remediation of PFAS-contaminated soils is critical for breaking this contamination cycle, protecting groundwater resources, and reducing long-term health risks associated with PFAS exposure. Thus, removal of PFAS from these sources becomes a compelling research topic. Currently, the main PFAS removal methods include chemical oxidation/reduction,^4^ photochemical,^5^ electrochemical processes,^6^ and thermal treatment.^7^ Among them, high-temperature degradation is a standard method, but it is prolonged and often degrades soil quality. We recently demonstrated a flash Joule heating (FJH) method that electrothermally degrades PFAS.^8,9^ The FJH process rapidly heats soil to >1000 °C within seconds. This technique achieves rapid and efficient PFAS destruction with a PFAS removal rate of >99% and a mineralization efficiency of ∼90% while preserving soil structure, composition, and biological function, thus offering a promising solution for soil remediation. Its scalability, reduced energy consumption, and minimal secondary pollution make it a highly attractive strategy for large-scale, sustainable soil remediation.

While FJH is efficient in degrading PFAS, the process is intrinsically difficult to understand because it involves a complex physical-chemical reaction mechanism and nonlinear coupling between the reaction parameters. The extremely brief reaction durations, spanning only milliseconds to seconds, complicate the accurate observation and analysis of transient intermediate states, substantially restricting effective experimental design and process optimization.^8,9^ Traditionally, in a human-centered experimental design, a researcher is the optimizer, exploring the optimization space intuitively and then conducting experiments via traditional trial-and-error methods.^10^ The pathway to reaching the global optimum is uncertain and largely depends on human knowledge and skills. Hence, accurately describing and optimizing the process theoretically through traditional methods becomes a formidable scientific challenge, thus opening new opportunities for emerging data-driven approaches.^11,12^ This is because, as the complexity of the optimization space significantly grows with the number of variables, a lack of knowledge about the experiments such as the one associated with FJH would make physics-based modeling ineffective. Recently, we employed a complementary data-driven approach, enabling the identification of hidden patterns in multidimensional chemical space for correlating the processing with the structures and properties.^13,14^ In particular, Bayesian optimization (BO), a verification-by-learning active learning (AL) framework, can build reliable models via iteratively querying unlabeled data.^15,16^ It permits rigorous uncertainty quantification and thus has been widely explored as an experiment planning strategy.^17–22^ While BO has gained popularity in evaluating scientific tasks because of its low data requirements, a vanilla BO algorithm has yet to fully gain trustworthiness due to its black-box nature. In contrast, human researchers can derive knowledge from discrete data and rare events. As a result, there has been much interest in involving humans in the BO loop. This approach is particularly applicable to scientific and exploratory tasks such as materials discovery.^23,24^ In the case of semiconductor process optimization,^25^ human involvement in the generation of candidate solutions in the early optimization phase, while allowing the model to fully undertake the optimization task as experimental data accumulates, facilitates the overall convergence of the model to achieve optimum results. While human-in-the-loop BO has shown considerable potential in accelerating scientific discovery, current frameworks typically rely on empirical strategies, offering limited capacity to elucidate underlying physical or chemical mechanisms.^26^ This shortcoming fundamentally constrains their applicability to complex, rapidly evolving reactions, such as FJH, where mechanistic insight is essential for a rational process design.

Herein, we propose a data-driven materials discovery workflow that includes a human-guided Bayesian Optimization (HGBO) algorithm and a multibranch neural network (MBNN) to understand and optimize PFAS removal from soil. In the HGBO algorithm, a scaled expert confidence score informed by intuition and visualization of the model’s posterior predictions based on the results from past experiments is incorporated into an acquisition function in each optimization iteration, enabling probabilistic, expert-guided experiment selection. This work is optimized within experimental constraints. While historical data are available, most of it originates from empirically guided exploration and exhibits a severely imbalanced distribution, resulting in limited effective coverage of the design space. It is within this context that, under experimental budget limits, the HGBO method achieved a significant improvement in perfluoroalkyl substance removal efficiency in just two iterations. Moreover, we elucidate underlying factors influencing PFAS degradation using interpretable model techniques, including SHapley Additive Explanations (SHAP) and Partial Dependence Analysis (PDA). These analyses clarify the feature importance and interactions, providing insights into how the model makes decisions. This interpretability substantially enhances the practical utility of the HGBO framework. In parallel to HGBO, we also developed a multibranch neural network that feeds chemical features, structure descriptors in SMILES, and flash experimental parameters, respectively, into three separate branches and then fused to predict the FJH efficiency. The SHAP values of these input variables were extracted and ranked to explore their effects on the PFAS degradation. This helps to identify key functional groups that affect the outcomes of PFAS removal by FJH, which were then validated by density functional theory (DFT) calculations.

## 2 Results and Discussion

### 2.1 Workflow of the Data-Driven Optimization for PFAS Removal

To configure precursor compositions, three additives, including biochar, carbon black, and metallurgical coke, were mixed with soil at varying ratios (Table S1). By analyzing 52 initial data sets obtained from flash Joule heating (FJH) experiments (Figure S1), we observed that only voltage was linearly related to PFAS removal, whereas the relationships involving other experimental parameters remain unknown (Figures S1 and S2). In addition, the historical experimental data are skewed, distributed across the parameter space as a result of experience-driven sampling (Figure S3). Together, this uncertainty and potential nonlinearity between multiple parameters describe the system as a high-dimensional black-box problem, necessitating the use of BO methods to efficiently determine optimal conditions. Therefore, the generated data were first used to pretrain a surrogate model in the BO pipeline (Figure 1a). Our HGBO incorporates a human-in-the-loop mechanism that dynamically guides the optimization trajectory in each batch. Specifically, for each candidate (experimental condition set) suggested by the acquisition function, a domain expert provides real-time feedback based on outputs of mean predictions, uncertainty, and sample distribution that can be directly visualized (Figure 1b). These human-assigned scores do not trigger model retraining but directly reshape the acquisition function, steering subsequent selections toward more informative and uncertain regions. This interactive strategy enhances sampling efficiency by reprioritizing GP means. After the suggested candidates in this present iteration are selected, they will be tested by experiments for the next iteration. The resulting data are then used to update the model used in the optimization algorithm. To further elucidate the mechanistic basis of PFAS degradation, we developed an MBNN that integrates optimized experimental data, molecular descriptors, and graph-based representations of four PFAS structures (Figure 1c and Table S2). The MBNN is used for mechanistic interpretation rather than as a surrogate model in HGBO because BO critically relies on well-calibrated uncertainty estimates and data-efficient exploration, which are more robustly provided by GP-based surrogates under the present data regime.^27^ SHAP analysis was used to assess the global contribution of these chemical features, while a perturbation-based masking strategy enables atom-level attribution, quantifying the relative importance of individual inputs to the predictive outcome. The comprehensive results, encompassing both molecular descriptor analysis and atom-specific importance rankings, were subsequently validated by DFT calculations (Figure 1d). In contrast to traditional human-centered trial-and-error methods, this proposed human-guided optimization workflow not only incorporates expert feedback to accelerate convergence but also introduces interpretability into the optimization process itself, providing insight into black-box decision-making. Furthermore, unlike transformer-based attention models that require large data sets to yield reliable atom-level explanations,^28^ our approach provides chemically meaningful and high-resolution interpretability in data-scarce settings through perturbation-based attribution integrated with a graph-aware architecture and expert-informed optimization.

**Figure 1:**
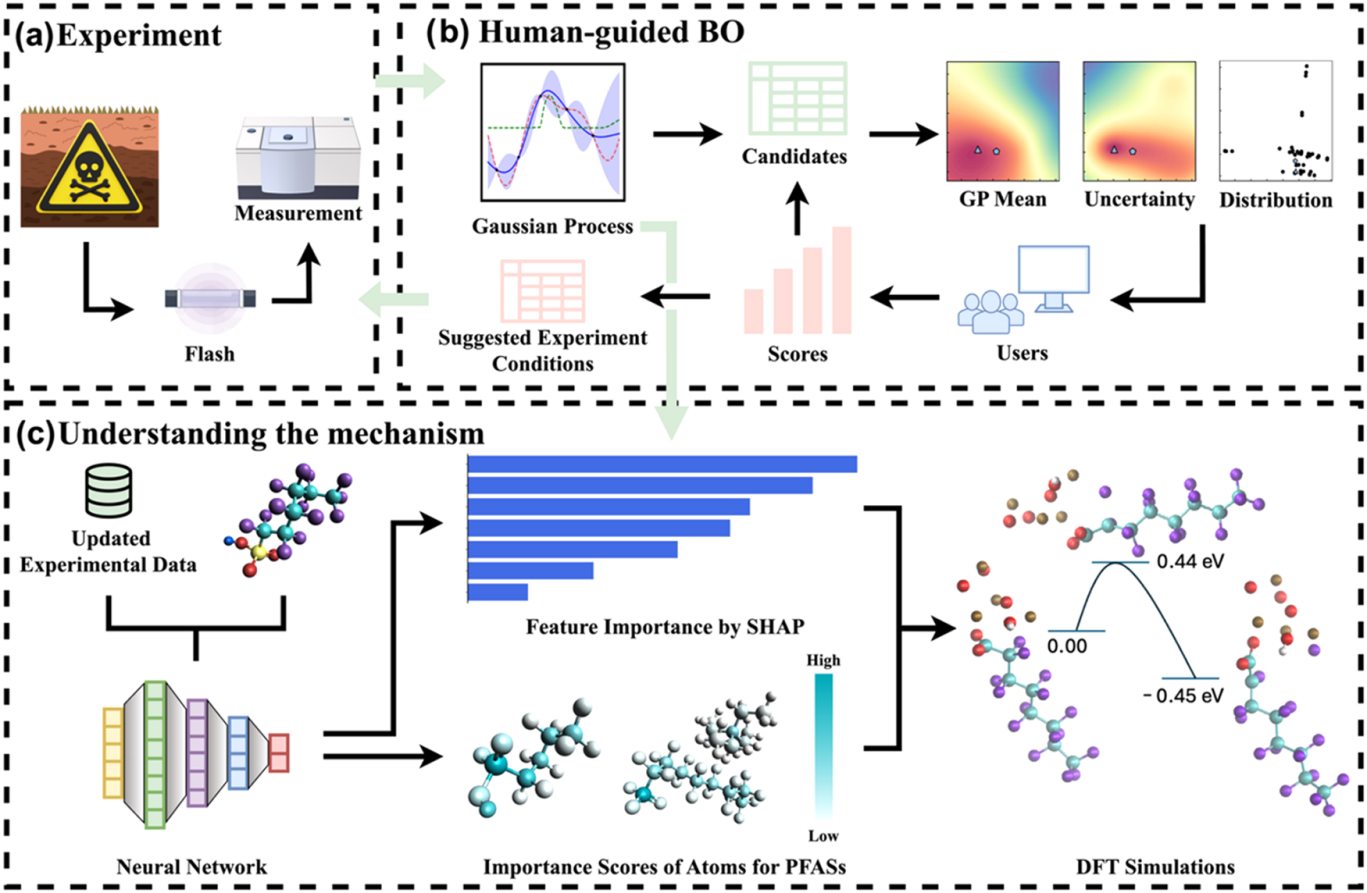
Overview of the data-driven optimization process for PFAS degradation by FJH. (a) Schematic of the FJH process. (b) Scheme showing a workflow of the HGBO algorithm. A human expert assesses the model-generated recommendations, assigns confidence scores, and decides the subsequent experiment. (c) Mechanistic understanding via SHAP feature attribution (GP and MBNN), atomic-level importance mapping from MBNN, and DFT-based validation.

### 2.2 Development of the Human-Guided Bayesian Optimization (HGBO) Algorithm

#### 2.2.1 Architecture of the HGBO Algorithm

The HGBO framework starts with user-defined input features, including precursor formulation descriptors (type of additive materials, additive ratio, and mass) and the voltage. Electrical resistance was predicted by an XGBoost model pretrained on historical data generated in the initial 52 experiments (Figure 2a). Resistance was included as a feature in the surrogate model due to its high SHAP attribution (Figure S4). By comparing the tree-based model (Random Forest, XGBoost) to other linear models (Bayesian Regression) (Figure S5 and Table S3), XGBoost results in an RMSE of 0.028 and an *R* ^2^ of 0.97, while the other two models show higher RMSE values and lower *R* ^2^ (Figure S6). Thus, it represents a better choice for the nonlinear transformation of precursor descriptors and serves as a stabilizing proxy for the system variability. In practice, even when the formulation is nominally identical, the resistance values can vary due to sample heterogeneity, packing inconsistencies, or instrumental noise. Including this predicted resistance in the surrogate model as a constraint (Supporting Method 2) helps buffer the experimental uncertainty, thereby improving the model’s robustness and generalization. A similar strategy has been used in our previous work.^14^ The predicted resistance, together with the precursor parameters and voltage, is then imported as input features to the GP, which predicts the remaining PFAS amount left in the soil after FJH. The GP posterior informs the acquisition function to suggest a candidate condition input for a new experiment (Figure 2a). These suggested inputs and the associated predicted outputs are projected onto a 2D space for direct visualization. Domain experts assign qualitative scores based on feasibility and scientific intuition. Selected candidates are experimentally tested, and the outcomes are fed back into the XGBoost and a GP model, completing the optimization loop. This iterative framework enables data-efficient exploration of a complex chemical parameter space under uncertainty for dynamically adjusting feature relevance as the model converges.

**Figure 2:**
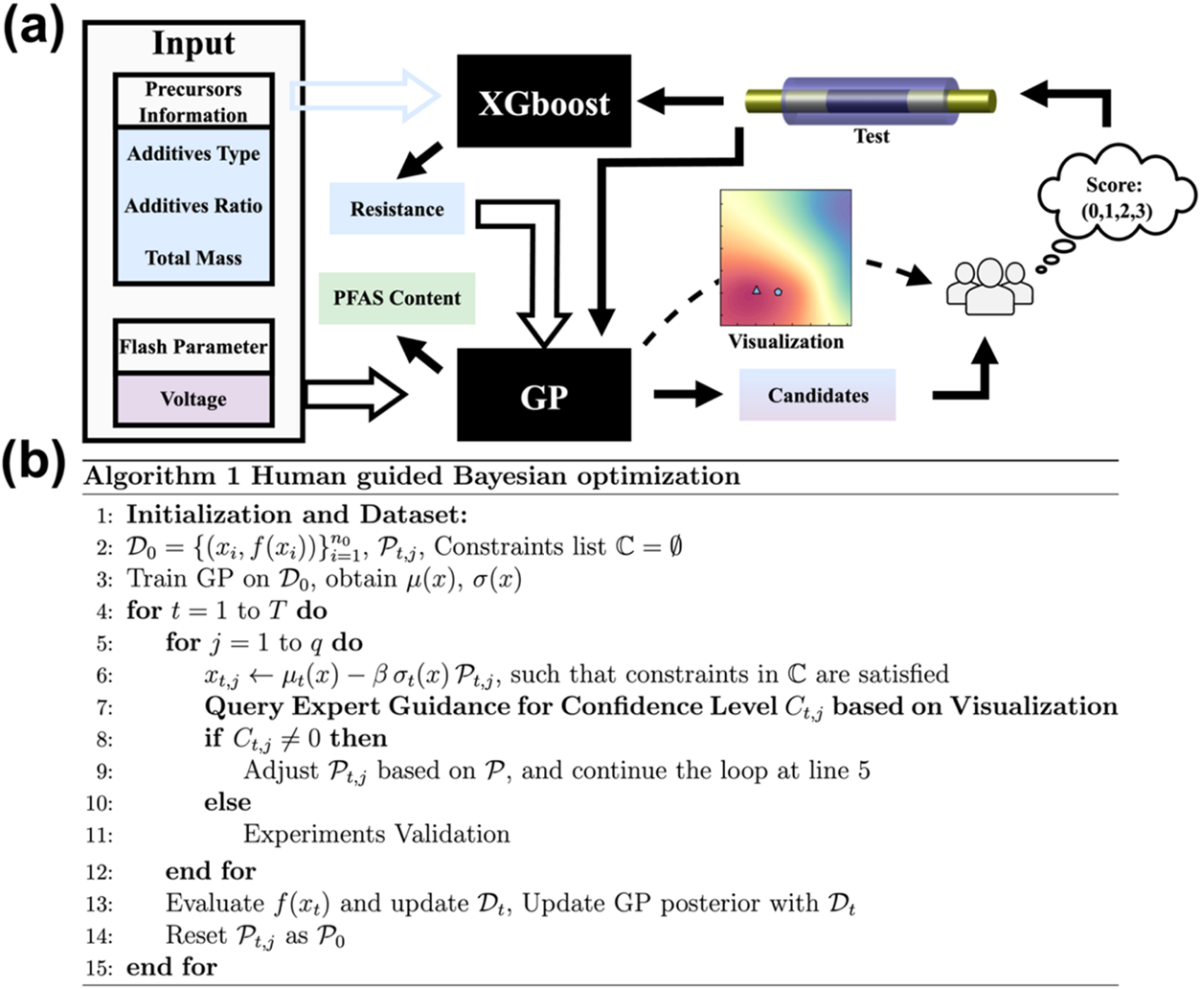
Overview and algorithm of HGBO for PFAS removal by FJH. (a) Scheme showing the dataflow in the HGBO framework. (b) Pseudocode for the HGBO algorithm, detailing expert intervention and feedback loops within the candidate selection process.

Compared to a vanilla BO,^15^ which solely relies on surrogate model uncertainty and fixed hyperparameters to navigate the search space, our HGBO introduces two key advantages. First, it overcomes the rigidity of fixed exploration-exploitation trade-offs by incorporating real-time expert feedback that reflects contextual interpretability. Second, it avoids common pitfalls of the vanilla BO, such as premature convergence or inefficient exploration, especially in small-data regimes with complex physicochemical interactions. By tightly integrating human intuition with model-driven inference, our approach not only accelerates convergence but also enhances the robustness and interpretability of the optimization process, an essential advantage in experimental domains like PFAS degradation, where experimental data are costly and interpretability is critical.

#### 2.2.2 Acquisition Function Used in the HGBO Algorithm

The pseudocode for the HGBO algorithm is illustrated in Figure 2b. A key innovation of this algorithm is the design of a dynamically updated acquisition function (AF). BO offers an efficient framework for the global optimization of a black-box function *f*(**x**), represented as

1 $$x^{*}={\mathrm{argmin}}_{x\in X}f\left(x\right)$$

where **x** corresponds to the search space. BO models the objective function *f*(**x**) by placing a prior distribution over the space of unknown objective functions and then uses it to determine the next best candidate for evaluation. Specifically, a GP is selected as a surrogate model. Based on the posterior mean and uncertainty provided by the GP, an AF is constructed to guide the selection of the next evaluation point. In our algorithm, a human guidance implemented AF is given by

2 $$\alpha_{t,j}\left(x\right)=\mu \left(x\right)-\beta_{t}\sigma \left(x\right)P_{t,j}$$

where β_*t*_ is an exploration weight and *P*_*t*,*j*_ is a probabilistic scaling factor derived from expert confidence with *j*th candidate at iteration *t*. The scaling factor is initialized as *P*_*t*,0_ = 1 at the beginning of each iteration and is updated recursively according to

3 $$P_{t,j}=P_{t,j-1}\cdot n^{g\left(C_{t,j}\right)}$$

where *n* > 1 is a hyperparameter that controls the amplification rate of confidence-driven exploration by bounding the per-candidate multiplicative factor to the range [1, *n*]. The confidence scores *C*_*t*,*j*_ ∈ {0, 1, 2, 3} represent expert assessment informed by the distribution of existing experimental data, the GP-predicted mean and uncertainty, and domain-specific intuition. The confidence score is mapped to a bounded monotonic modulation factor *g*

4 $$g\left(C_{t,j}\right)=\frac{1}{1+\exp\left(-6\left(C_{t,j}-1.5\right)\right)}\;\epsilon \;\left[0,1\right]$$

The sigmoid mapping *g* converts the discrete confidence score into a continuous modulation term that scales the uncertainty contribution in the AF (Figure S7). The slope and inflection point of the sigmoid function in eq 4 are set to 6 and 1.5, respectively (details in Supporting Method 3). To initiate the confidence scoring process, we first visualized the distribution of candidates relative to the previously collected experimental data by projecting them into two-dimensional space using principal component analysis (PCA) (Figure 3a). This projection allows for an intuitive qualitative assessment of whether the candidates are located within a known design region (Candidate 1 in interaction 1) or are exploring a previously untested region (Candidate 2 in interaction 1). Candidates located near clusters of historical data are assumed to be within the already explored regions of design space, while those located farther away are likely to be in uncharted territory. However, because PCA compresses complex high-dimensional data into a reduced representation, it may obscure nonlinear boundaries or subtleties that are critical to decisions in the original input space. Thus, while the PCA projection provides a useful overview of the model’s exploration-exploitation behavior, it alone is insufficient to support reliable expert judgment. To address this limitation, the predicted GP mean and uncertainty as a function of the two reduced principal features of the candidate are also extracted and then visualized in Figure 3b,c, respectively. If the GP predicts a high mean and low uncertainty for a recommended candidate, meanwhile being close to the historical data cluster in the PCA projection (e.g., Candidate 1 in interaction 1), the candidate is assigned as lying within a well-explored region of the design space. In this case, the human expert has high confidence in the surrogate model’s prediction, and the confidence score is assigned as *C*_*t*_ = 3. A score of 3 indicates that the recommendation is consistent with prior experimental experience and therefore does not require immediate experimental validation; instead, it is used to inform subsequent acquisition weighting without being executed. At an opposite extreme, when a recommended candidate is far from the historical data cluster (e.g., Candidate 2 in interaction 1), it is considered to be outside the region covered by prior experimental observations. In this situation, the confidence score is assigned as *C*_*t*_ = 0, indicating that the candidate represents a substantially new region of the design space. *C*_*t*_ = 0 prioritizes experimental validation, reflecting a deliberate strategy to acquire new information in that region. Candidates that fall between these two extremes are assigned intermediate scores. In this intermediate regime, *C*_*t*_ = 2 is assigned when the expert determines that the subsequent iteration should prioritize exploration, whereas *C*_*t*_ = 1 is assigned when the expert prefers more exploitation search behavior. These confidence scores are embedded into the AF through a dynamic weighting mechanism (eq 3), which adjusts the exploration–exploitation balance in subsequent iterations. This feedback mechanism ensures that if a higher expert confidence happens, the model will favor exploration, whereas low confidence drives the algorithm toward exploitation or experimental validation.

**Figure 3:**
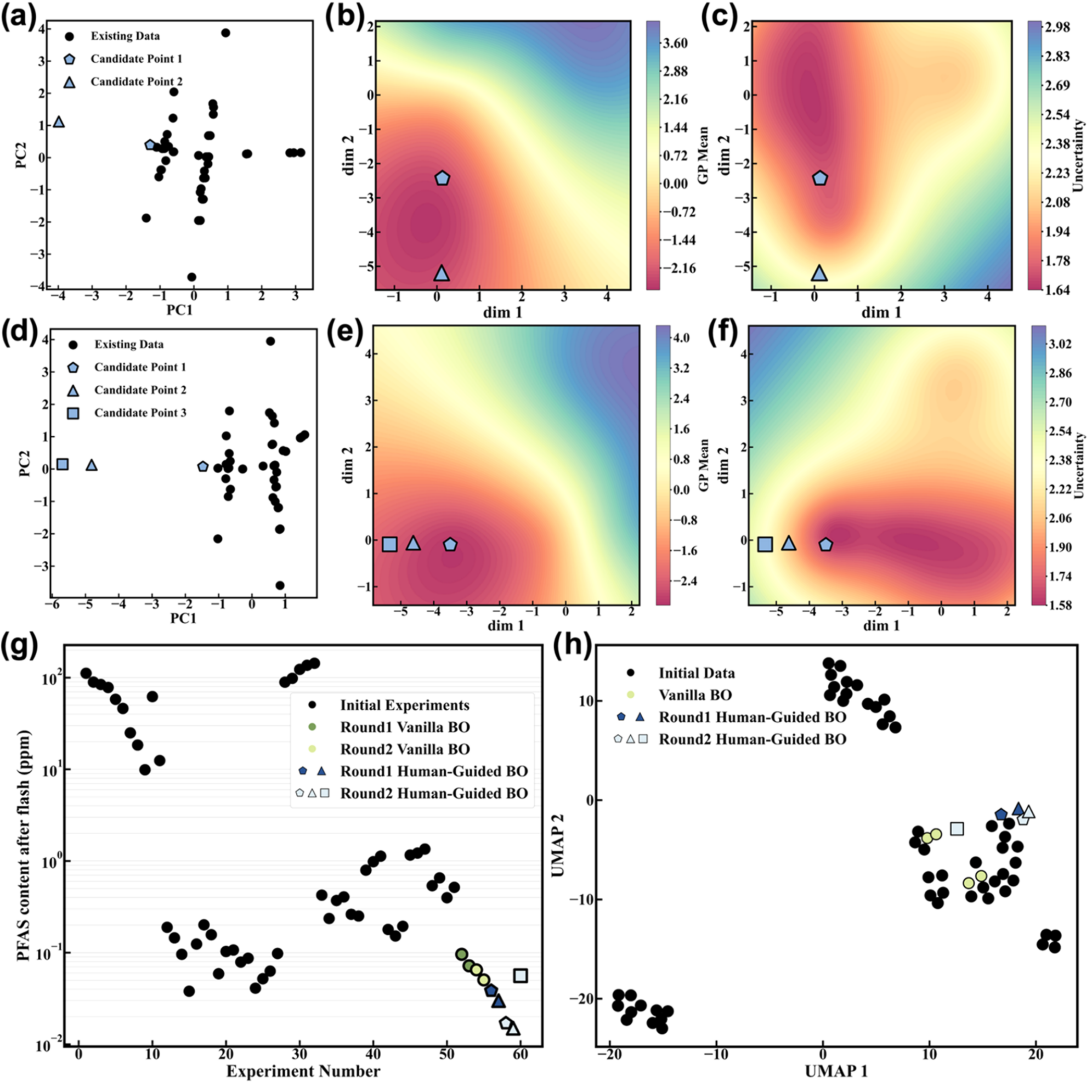
Visualization and performance of HGBO. Decision-making across two subsequent iterations: (a–c) Iteration 1; (d–f) Iteration 2. Visualization of candidate points in PCA plots (a, d), GP mean predictions (b, e), and GP uncertainty (c, f). (g) Evolved PFAS content afforded by human optimization, vanilla BO, and HGBO algorithms. (h) UMAP projection of experimental inputs. Each point is a distinct experiment. Standard BO samples cluster in dense regions; HGBO explores boundaries and sparse zones.

It is important to emphasize that the role of the confidence score in HGBO is not to introduce an alternative metric for exploration nor to replace conventional uncertainty-based acquisition. Instead, it provides an operational decision rule that bridges the model uncertainty and experimental action. In a vanilla BO, increasing the exploration parameter (β) indicates a preference for uncertain regions, but it does not explicitly determine when experimental validation becomes necessary, or which uncertain candidate should be executed under a limited budget (Table S4). In contrast, the HGBO formalizes this step by prioritizing the unfamiliar candidates to trigger experimental validation without reasoning about abstract acquisition parameters. By externalizing this decision into a visualization-guided confidence assignment, HGBO replaces opaque parameter tuning with a stable and reproducible experimental decision process that is aligned with how exploration is conducted in practice.

#### 2.2.3 Decision Process and Experimental Outcomes

To systematically evaluate the decision-making process and optimization efficiency of HGBO, we visualized candidate selection and experimental outcomes across two iterations (Figure 3a–f). In the first iteration (Figure 3a–c), two candidate points were selected through expert evaluation of model predictions and associated uncertainties, guided by the AF, as previously described. In the second iteration (Figure 3d–f), the same evaluation criteria were employed to select three additional candidates, further promoting exploration beyond previously sampled regions. Notably, Candidate 3 selected in the second iteration highlights a defining aspect of HGBO. Candidate 3 is located far from existing data points in the PCA embedding (Figure 3d), presenting a significant challenge for expert evaluation due to the lack of intuitive or prior knowledge about this unknown region. The evaluation results of the acquisition function for each candidate in each iteration are detailed in Table S5.

The goal of this HGBO algorithm is to reach as low-PFAS content as possible in the soil by FJH within as few iterations as possible. The PFAS contents of the initial 52 experiments performed by a human expert and the PFAS contents afforded by two iterations of the HGBO algorithm are shown in Figure 3g. Among the initial 52 experiments, the lowest PFAS content that our human expert can achieve is 0.038 ppm. Using HGBO, the PFAS concentration was reduced to 0.015 ppm within two iterations, corresponding to an approximately 60% reduction. An examination of the historical sampling landscape shows that low-PFAS outcomes in the initial data set are strongly clustered within a narrow region of the parameter space. Most prior experiments are concentrated at the precursor weight ratio of 2:1 and the voltage of 100 V, where repeated experimentation yielded a plateau without further improvement (Figure S3a–f). Notably, candidate conditions recommended by vanilla BO are also largely clustered within this same region (Figure 3g and Tables S4 and S6). In contrast, the HGBO recommended conditions that resulted in 0.015 ppm PFAS are outside this historically concentrated region. This indicates access to an experimentally feasible processing regime that was not reached through prior intuition-led exploration. The associated shift in experimental coverage is further reflected in a two-dimensional Uniform Manifold Approximation and Projection (UMAP) embedding of the experimental parameter space (Figure 3h and Tables S4–S7), where the recommended conditions extend toward the periphery of dense historical clusters and into sparsely sampled regions. It suggests that PFAS degradation under FJH depends on specific combinations of processing parameters rather than monotonic trends in individual variables. Our HGBO can effectively explore beyond historically favored configurations for more efficient PFAS destruction.

#### 2.2.4 Model Interpretation to Understand the FJH Mechanism

This task aims to understand how the algorithm correlates the input features with the output predictions. To assess each feature’s impact, their SHapley Additive exPlanations (SHAP) values were calculated and analyzed. A higher SHAP value suggests a higher impact of this feature in determining the output. Figure 4a illustrates the SHAP values of each feature. It shows that voltage has the greatest impact, characterized by a wide range of SHAP values in both positive and negative directions. Specifically, higher voltages (red data points) correspond to higher positive SHAP values, indicating its substantial impact in reducing the PFAS content. Conversely, a lower voltage (blue data points) is associated with negative SHAP values, reflecting suppressed reactivity in the presence of the lower voltage. The additive ratio and additive material follow voltage show their high weights in shaping model predictions (Figure 4a,b). This is because additives (such as biochar) can be chemically involved in the defluorination process by releasing reactive substances such as Ca^2+^ that promote PFAS mineralization.^8^

**Figure 4:**
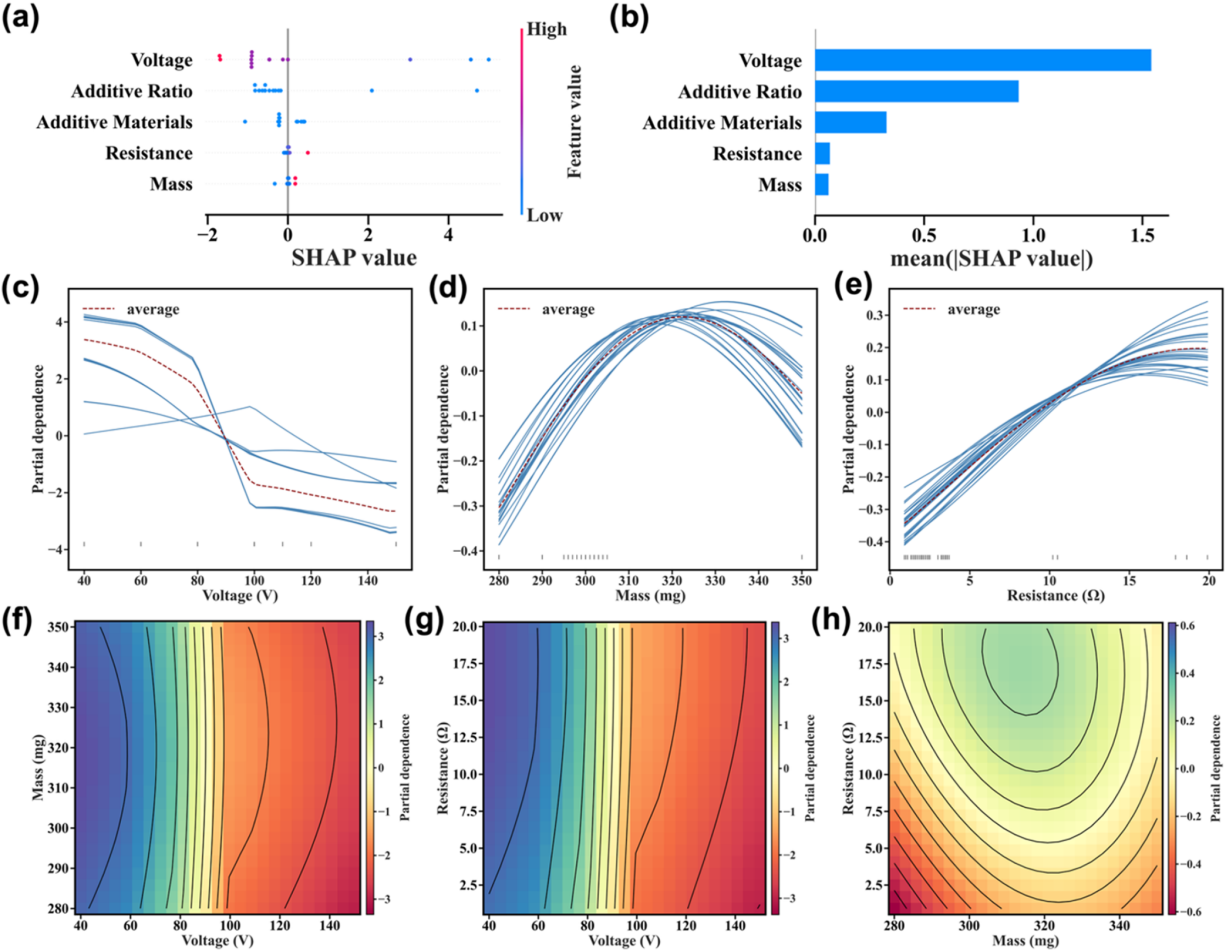
Feature importance and interaction analysis on HGBO. (a) Relative SHAP value distribution for each feature, colored according to feature importance. (b) Average absolute SHAP values. (c–e) PDP illustrating the isolated effect of individual key features on predictions: (c) Voltage; (d) mass; (e) resistance (Ω). (f–h) 2D PDP contour maps showing interactions between key features: (f) Mass vs voltage; (g) resistance vs voltage; (h) resistance vs mass.

To gain mechanistic insights into how individual and interacting features shape the model’s prediction, we conducted both one-dimensional (1D) and two-dimensional (2D) partial dependence analyses (PDA) (Figure 4c–h). Notably, since the optimization target is to minimize the output, negative partial dependence values are favorable. In Figure 4c, voltage exhibits a consistently negative influence on the prediction, with the most pronounced effect observed between 60 and 120 V. This indicates that increasing voltage within this range is strongly associated with reduced predicted values, consistent with SHAP results that the voltage is ranked as the most impactful feature. Such a relationship may arise from enhanced thermal degradation efficiency under higher electric field strengths.^8,29^ In contrast, mass shows a nonmonotonic impact (Figure 4d). As the mass increases from ∼280 to ∼320 mg, the predictions increase, suggesting an unfavorable effect in this region. However, beyond ∼320 mg, the predicted values begin to decrease, suggesting that larger sample masses may enhance heat retention or field distribution, thereby improving degradation at higher loads. This peak behavior may be explained by a balance between heat/mass transfer efficiency and total energy absorption. Resistance shows a monotonic positive correlation with the prediction (Figure 4e), indicating that higher resistance hinders the desired minimization outcome. However, its overall contribution to model predictions is relatively minor compared with the voltage and additive ratio, as reflected in the SHAP summary (Figure 4a,b). The two-dimensional (2D) partial dependence plots (PDP) further reveal feature interactions. The voltage–mass map (Figure 4f) highlights the red region of low prediction values at high voltage and low mass (∼280 mg), consistent with the 1D trends. A similar red region is seen in the voltage–resistance map (Figure 4g), where optimal outcomes arise under high voltage and low resistance (∼5 Ω). Interestingly, the mass–resistance map (Figure 4h) indicates a subtle saddle-shaped interaction: while increased mass and decreased resistance individually lower the prediction, their combined effect saturates near a specific region, suggesting diminishing returns. These findings underscore that optimal performance emerges not solely from any single variable but from its finely balanced interactions.

### 2.3 Understand the PFAS Removal Mechanism by MBNN and DFT

Besides the analysis of the features to understand the trained GP model, we trained an interpretable ML model to probe the microscopic, structure-specific determinants. Therefore, we adopted a multibranch neural network architecture (Figure 5a) consisting of chemical features, molecular structure, and experimental process parameters. In this case, we compiled the data from four types of PFAS: PFBS, PFHxS, PFOA, and PFOS. The chemical features branch ingests molecular descriptors (Sulfonic Group (−SO_3_), Carboxyl Group (−COOH), Labute approximate surface area (LabuteASA), average atomic weight (AAW), max partial atomic charge (MPAC), number of rotatable bonds (NRB), molecular weight (MW), and heteroatom count (HC)) and transforms them through multiple fully connected layers to produce high-level chemical embedding. In the branch of the molecular structure, each atom of each PFAS is encoded individually, and its relative position and local environment in the molecular structure are captured by a graph neural network in conjunction with its connectivity relationships. The branch of experimental data uses the same hidden stack to process the experimental parameters, including voltage, resistance, and mass to produce processing embedding. These three embeddings are then merged in a fusion block to the final layer to map the fusion representation to a single output for the PFAS content prediction. To quantitatively evaluate the performance of MBNN, we performed regression analysis on the data set of four representative types of PFAS processed under diverse experimental conditions. As shown in Figure 5b, cross-validation results demonstrate close correspondence between predicted and measured removal efficiencies, yielding an average *R* ^2^ of 0.95 ± 0.02 and RMSE of 0.62 ± 0.17 (transformed units) across folds. Compared to previous approaches where chemical descriptors and process conditions were treated as a unified input,^30^ the MBNN architecture can explicitly distinguish between chemical and processing features for independent feature extraction and high-level fusion,^31^ thus enhancing the model’s ability to capture the distinct impacts of the chemical and processing features on the PFAS removal efficiency.

**Figure 5:**
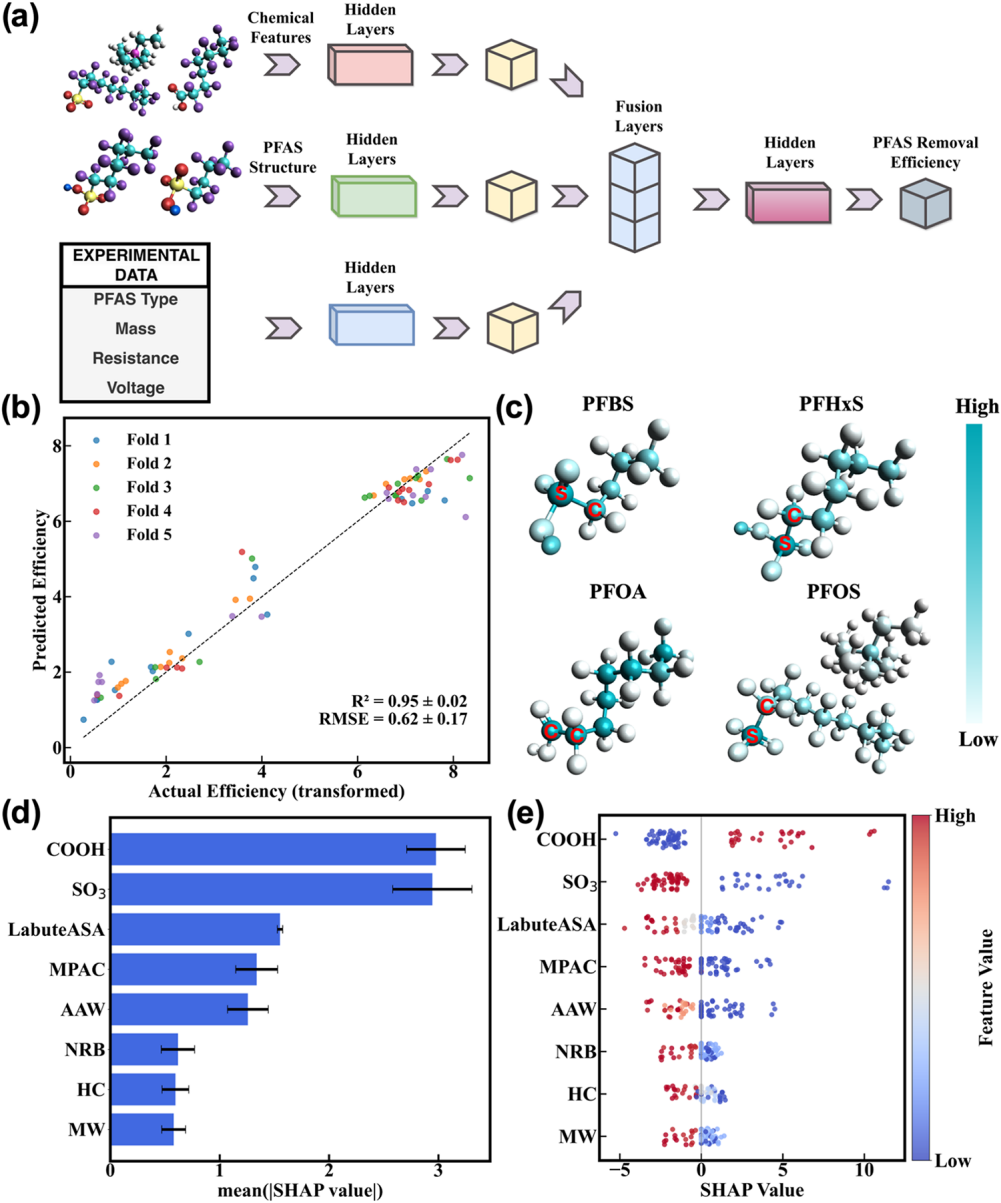
MBNN and feature analysis to understand PFAS FJH removal. (a) Scheme of the MBNN architecture. (b) Cross-validation parity plot of predicted versus actual transformed efficiency. Colors denote individual folds, and the dashed line represents perfect prediction (*y* = *x*). (c) Atom-level attribution score maps highlighting the importance of functional groups. For PFBS, PFHxS, and PFOS, the atoms assigned the greatest importance are sulfur and carbon, whereas for PFOA, the most important atoms are carbons within the molecule (highlighted in red). (d) Global SHAP importance of 8 chemical descriptors under cross-validation. Bars show the average absolute SHAP magnitude across folds, and error bars denote ±1 standard deviation across outer CV folds. (e) SHAP beeswarm plot of local SHAP values for the 8 most important chemical descriptors. Each point corresponds to one sample, and the vertical spread reflects the density of SHAP contributions.

To assess which molecular substructures are prioritized by the MBNN, we performed an atom-level perturbation analysis on the graph branch, in which each atom’s feature embedding was masked individually, and the resulting change in the graph-level output was used to quantify its importance. This approach enables the generation of atom-level attribution maps for each PFAS (Figure 5c). Details of the individual atom scores for the four PFAS are shown in Tables S8–S11 and Figure S9. In these visualizations, atoms with higher attribute weights are highlighted, reflecting their greater influence on the removal outcome. Notably, atoms within the sulfonic acid groups (like the ones in PFOS and PFBS) consistently receive the highest attribution score, whereas carboxyl group-containing sites (like the ones in PFOA) are also strongly weighted, but with distinct spatial distributions. Hence, sulfonic and carboxylic acid groups are identified as the main factors that promote the PFAS removal. Figure 5d,e presents the summary analysis of cross-validated SHAP values, indicating that feature effects remain consistent across different folds. The carboxyl group exhibits the highest negative contribution to predicting degradation efficiency, while the sulfonate group ranks second with a positive contribution. Since all four PFAS compounds in the training set include either a sulfonic acid or a carboxyl group, these opposing effects indicate that PFAS with carboxyl groups degrade more readily compared to those with sulfonic acid groups. Interestingly, the correlation between the carbon chain length and model predictions was weak. This observation is consistent with the thermal degradation behavior of perfluoro carboxylic acids, where chain length does affect decomposition stability, but the impact of functional group identity dominates the overall thermal stability trend.^7^

To further corroborate the multiscale feature importance identified by the feature analysis and the atom-level attribution map, we conducted DFT calculations to quantify the defluorination barriers of representative PFAS structures catalyzed by CaO (Figure 6). In these calculations, the PFCA class was used as the representative structure for PFOA, while two PFCS structures with different chain lengths were selected to represent PFOS, PFHxS, and PFBS. Long- and short-chain PFCA species exhibit activation energies of 0.44 and 0.61 eV, respectively, with exothermic reaction enthalpies of −0.45 and −0.55 eV (Figure 6a,b). By contrast, the analogous PFCS systems (Figure 6c,d) require substantially higher barriers of 0.84 and 0.82 eV and display near-thermoneutral enthalpy changes (0.03 and 0.02 eV). Although chain extension raises the PFCA barrier by 0.17 eV, this increment pales in comparison to the ∼0.40 eV disparity between PFCA and PFCS, and the 0.10 eV enthalpy shift falls within typical DFT uncertainties (±0.1–0.2 eV).^32^ These DFT calculations confirm that the carboxyl group promotes defluorination by lowering the kinetic barrier while the sulfonate group hinders degradation by raising the activation threshold. Hence, the modest chain-length dependence for PFCA and its near absence in PFCS mirror the minimal SHAP importance assigned to the carbon chain length. These DFT results agree well with the analysis in Figure 5c–e, suggesting that our developed MBNN can catch the chemical insight well.

**Figure 6:**
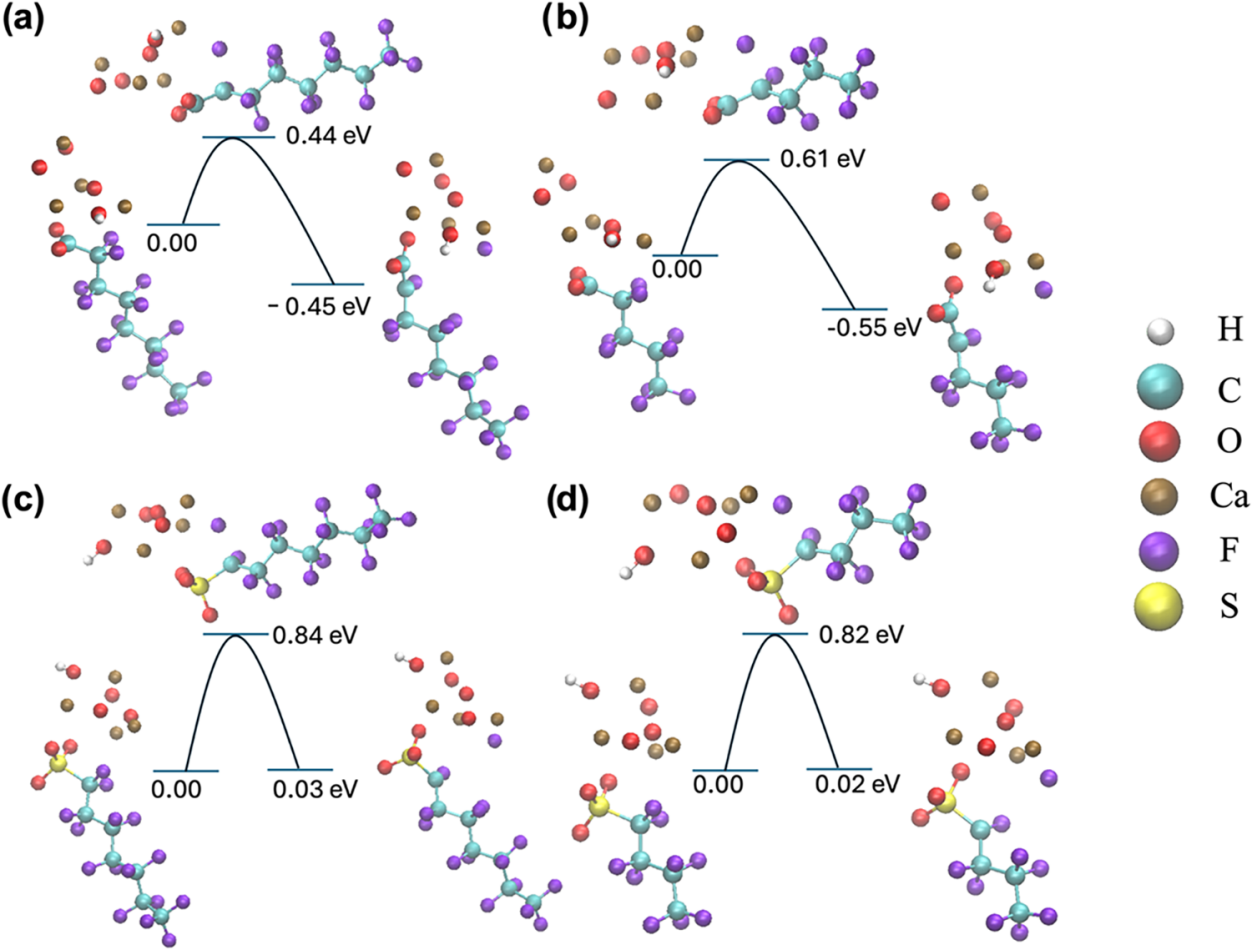
DFT calculation on kinetic barriers to defluorination of PFCA and PFCS catalyzed by CaO. Energy calculations on PFCA with long (a) and short (b) carbon chains. Energy calculations on PFCS with long (c) and short (d) carbon chains. C, F, O, H, Ca, and S are shown in cyan, purple, red, white, ocher, and yellow colors, respectively.

## 3 Conclusion

We present a data-driven optimization framework for FJH removal of PFAS from soil by combining HGBO and MBNN. Building on the existing experimental data set and under a limited number of additional experimental iterations, the HGBO workflow achieves a 60% reduction in residual PFAS concentration within two optimization iterations relative to the previously best observed outcome. In the HGBO strategy, expert input is incorporated into experiment selection through structured guidance supported by visualization of the model predictions and the underlying input space. Owing to its interpretability and its ability to operationalize domain knowledge during experiment planning, HGBO is broadly applicable to optimization problems characterized by complex, high-dimensional parameter spaces and costly evaluations, including materials processing and chemical reaction systems. In addition, the MBNN extends our understanding from a single PFAS to multiple PFAS compounds at the microscale, revealing fundamental insights into molecular-level transformations. Complementary DFT calculations further elucidated the underlying reaction mechanism, demonstrating that functional groups rather than chain lengths play a dominant role in the defluorination process. Overall, this iterative, collaborative framework will enable efficient and informed exploration of the experimental space. It can be widely applied to optimization problems in broader disciplines where data are scarce and experiments are highly complex and costly.

## 4 Materials and Methods

### 4.1 Materials

Biochar (Wakefield Biochar, sourced from Amazon) was pretreated prior to soil mixing by applying a rapid electrothermal process at 60 V for 1 s, ensuring sufficient conductivity for its use as a conductive additive. Additional conductive materials, including carbon black (Cabot, Black Pearls 2000, ∼10 nm average diameter) and metallurgical coke (metcoke, SunCoke Energy), were also utilized. The PFAS compounds studied comprised perfluorooctanoic acid (PFOA, 95%, MilliporeSigma), heptadecafluorooctanesulfonic acid tetraethylammonium salt (PFOS, 98%, MilliporeSigma), tridecafluorohexane-1-sulfonic acid potassium salt (PFHxS, 98%, MilliporeSigma), and nonafluorobutane-1-sulfonic acid potassium salt (PFBS, 98%, MilliporeSigma). The raw soil was classified as a sandy loam. Clean soil was collected from the Rice University campus, crushed using a hammer grinder (Wenling LINDA Machinery Corporation, model DF-15), and oven-dried at 100 °C for 2 h to eliminate residual moisture. This soil was employed as a controlled model matrix to reduce variability associated with the moisture content and particle size. Accordingly, the experimental results reported in this study can be directly applied to soils with similar physicochemical characteristics. The environmental relevance of FJH-based PFAS remediation under more heterogeneous and moisture-containing soil conditions has been reported in previous studies.^8^

### 4.2 PFAS Content Measurement

A 200 mg portion of PFOA-contaminated soil and REM-treated soil was each dispersed in 2 mL of ultrapure water (MilliporeSigma, ACS grade for ultratrace analysis) at a soil-to-water mass ratio of 1:10. The suspensions were agitated on a horizontal shaker (Burrell Scientific Wrist Action, Model 75) for 2 h to ensure a thorough extraction. After shaking, samples were centrifuged (Adams Analytical Centrifuge) at 600 rpm for 2 min, and the supernatant was filtered through a poly­(ether sulfone) (PES) membrane (0.22 μm pore size, MilliporeSigma). Quantification was performed using a triple quadrupole LC/MS system (6740B LC/TQ, Agilent) operating in dynamic multiple reaction monitoring (DMRM) mode. Chromatographic separation was achieved with a C18 column (Zorbax Eclipse Plus C18 RRHT, 2.1 mm × 50 mm, 1.8 μm particle size, Agilent) on an ultrahigh-performance liquid chromatography (UHPLC) system (1290 Infinity II, Agilent). The mobile phases consisted of 20 mM ammonium acetate (aqueous) and methanol (organic), with a flow rate of 0.4 mL min^–1^ and column temperature maintained at 40 °C. Each injection used 40 μL of the sample. An Agilent Jet Stream (AJS) electrospray ionization (ESI) source, operated in negative mode, was coupled to the mass spectrometer. Calibration curves were prepared by using known PFOA concentrations in the range of 0.5 and 100 ppb. For analysis of other PFAS (PFOS, PFHxS, PFBS) by LC-MS (Figure S10), extract solutions were diluted with 90 vol % ultrapure water and 10 vol % methanol (HPLC grade, >99.9%, MilliporeSigma) to achieve target concentrations within the 0.5–100 ppb detection range.

### 4.3 Model Parameters and Interpretation

In each iteration, the GP was refined by incorporating newly collected data sets from the experiments into the database. To optimize the GP hyperparameters, we applied a global optimization of the log marginal likelihood following Schweidtmann et al.^33^ For the kernel function, we selected a composite kernel structure, a combined approach that simultaneously captures the global bias, local smoothness, and noise components of the data, thus improving the model’s ability to adapt to complex data structures.^34^

For the development of the MBNN model to predict the PFAS removal efficiency under flash Joule heating conditions, we integrated three distinct data modalities through dedicated input streams. These include an experimental branch for process-related variables, a branch for chemical features encoded by RDKit, and a graph branch that encodes molecular structure using graph attention networks (GAT).^35^ Each input stream is first processed independently through a series of fully connected or graph convolutional layers to extract the latent representations. These three representations are then concatenated and passed through a shared fusion module, followed by a regression head that outputs the predicted PFAS removal. The model was trained in a supervised manner using experimentally measured removal values, with the performance evaluated on a held-out test set. Details regarding feature processing, model architecture, training procedures, and interpretability analyses are provided in Supporting Method 3. The performance of the models was assessed by the coefficient of determination (*R* ^2^) and the root mean squared error (RMSE). To evaluate the performance of the MBNN, a 5-fold cross-validation (CV) was performed over the entire data set. When evaluated in the original efficiency space (0–100%), the model achieved a mean *R* ^2^ of 0.78 ± 0.09 with an RMSE of 8.73 ± 3.67 percentage points (Figure S11a), which is affected by the highly imbalanced data distribution (Figure S11c). To address this issue, the target efficiency was transformed using monotonic log mapping, as shown in eq 5.

5 $$y_{t}=-\ln\left(1-\frac{y}{100}\right)$$

CV in the transformed space yields substantial improvement (Figure S11b), with a mean *R* ^2^ and RMSE of 0.95 ± 0.02 and 0.62 ± 0.17 (transformed units), indicating that the model has learned a stable input–output relationship. The transformed and original evaluations were carried out using the same MBNN architecture and identical training hyperparameters, and the corresponding data distribution is shown in Figure S11d.

After training the models, we used SHAP, a commonly used XAI technique,^36^ to interpret the results afforded by the GP. The contribution of each variable to the model prediction was calculated by considering all possible variables.^37^ Thus, the use of SHAP allowed us to reveal the key factors in PFAS optimization. In addition, we performed a partial dependence analysis (PDA) to further elucidate the marginal effects of individual features and their interactions on model predictions. The partial dependence plots (PDPs), introduced by Friedman^38^ quantifies the expected prediction of a model as a function of one (or two) selected features. 1D PDPS illustrate how the predicted outcome changes as a single variable varies, while holding other variables constant. 2D PDPs extend this approach to visualize the interaction between pairs of variables.

### 4.4 Dimensionality Reduction for Visualization

Principal component analysis (PCA) was employed within the HGBO to project a high-dimensional parameter space onto a 2D subspace. The linear nature of PCA preserves the global variance structure and allows for interpretable, distance-preserving visualization of experimental data and recommended candidates. This facilitates the straightforward assessment of relationships and spatial proximity between candidate points and existing experiments, which is essential for human evaluation. To compare the overall distribution, clustering, and coverage of the explored parameter space across all optimization strategies, uniform manifold approximation and projection (UMAP) was applied to the entire data set.^39^ As a nonlinear manifold learning technique, UMAP is particularly effective at capturing complex local and global structures within high-dimensional data. Its use enables intuitive and persuasive visualization of the initial experiments and those by vanilla BO and HGBO, thereby revealing clustering patterns and the breadth of exploration achieved by each approach.

### 4.5 Density Functional Theory (DFT) Simulation

To simplify the inherently complex FJH process, we analyze a thermodynamically most probable reaction pathway to provide molecular-level insight. Thermodynamic analysis (Figure S12) shows that CaCO_3_ and Ca­(OH)_2_ become unstable and favor conversion to CaO within the FJH-relevant temperature window (∼1000–1500 °C), supporting the use of CaO as a high-temperature representative Ca phase. Defluorination of a PFCA molecule with a carboxylic head (−COOH) and a PFCS molecule with a sulfonic head (−SO_3_H), catalyzed by Ca^2+^ ions, is studied using the density functional theory (DFT) method as implemented in the Vienna Ab-initio Simulation Package (VASP). A plane wave expansion up to 520 eV is employed in combination with an all-electron-like projector augmented wave (PAW) potential.^40^ Exchange-correlation is treated within the generalized gradient approximation (GGA) using the functional parametrized by Perdew–Burke–Ernserhof.^41^ Because of the large supercell (a vacuum box of 18.0 Å × 18.0 Å × 25.0 Å containing a PFCA (or PFCS) molecule with four CaO molecules on its head), only the Γ point is used for the Brillouin zone integration over Monkhorst–Pack type mesh.^42^ In structure optimization using the conjugate-gradient algorithm, as implemented in VASP, the positions of atoms are fully relaxed so that the maximum force on each atom is smaller than 0.02 eV/Å. Defluorination of PFCA (or PFCS molecule) with either a longer carbon chain is compared with that with a shorter C chain. Kinetic barriers to the defluorination are studied using the climbing-image nudged-elastic-band method^43^ with nine images being used.

## Supplementary Material



## Data Availability

The used data set and codes are available at https://github.com/linresearchgroup/HGBO.

## Notes

Features distribution, simulated temperature of reactions inside the quartz tube, ML models’ hyperparameters, starting materials’ physical properties (PDF)
