# Supplementary material for: Accelerate Flash Removal of PFAS from Soil by Human-Guided Bayesian Optimization and Interpretable Machine Learning
Source: ACS Nano. 2026 Mar 23;20(13):10498–509. doi: 10.1021/acsnano.5c20063 (PMC13063811; doi:10.1021/acsnano.5c20063)
Supplement: Supplementary file 1 [file nn5c20063_si_001.pdf]

Supplementary Information for

Accelerate Flash Removal of PFAS from Soil by Human-guided Bayesian Optimization and Interpretable Machine Learning

Jingbo Qin[1#], Yi Cheng[2#], Malinda Jayathilake[1], Yufeng Zhao[5*], James M. Tour[2,3,4*], Jian Lin[1*]

[1]Department of Mechanical and Aerospace Engineering, University of Missouri, Columbia, MO 65211, United States
[2]Department of Chemistry, [3]Department of Materials Science and NanoEngineering, [4]Smalley-Curl Institute, NanoCarbon Center and the Rice Advanced Materials Institute, Rice University, Houston, Texas 77005, USA
[5]Corban University, 5000 Deer Park Drive SE, Salem, Oregon 97317, USA

[#]Authors equally contributed to the work.

[*]**Email**: linjian@missouri.edu; tour@rice.edu; yzhao@corban.edu

Table of Content

3 Supplementary Methods

8 Supplementary Figures

9 Supplementary Tables

5 Supplementary References

Supplementary Method

1. Input and output data analysis

Due to the large range of variations in PFAS content in the initial data (from 0.040 to 100 ppm), logarithms were used in both data analysis and training models to better demonstrate their distribution. We hypothesize that one of the main directions in BO is to explore around 1:1 weight ratio. As shown in Supplementary Fig. 2a-b, where the 1:1 weight ratio exhibits the lowest median Log PFAS Content and a relatively compact distribution, suggesting that this condition may be more effective in reducing PFAS levels. In contrast, the 2:1 and 3:1 weight ratio show higher variance in Log PFAS content, indicating that these ratios may lead to more unstable or higher PFAS levels. The 4:1 ratio, while showing a narrow distribution, is centered at a higher Log PFAS Content, suggesting that increasing the ratio beyond a certain threshold may not be beneficial for PFAS reduction. Despite this small mass variation, the log PFAS content spans over six orders of magnitude (from around –4 up to +4), indicating that within this mass window there is no clear linear trend between mass and treatment efficacy (Supplementary Fig. 2c). Supplementary Fig. 1d shows that for resistance >5 Ω, PFAS content remains high with little variation, but for resistance <5 Ω, the relationship becomes highly nonlinear and unpredictable, with PFAS degradation efficiency varying significantly. This indicates a black-box problem where simple linear models or traditional optimization methods fail. Bayesian optimization is necessary as it efficiently explores such complex, non-monotonic spaces, balancing exploration and exploitation to identify optimal conditions. We analyzed the relationship between voltage and PFAS content to identify trends that could guide Bayesian optimization for minimizing PFAS contamination. The scatter

plot (Supplementary Fig. 2e) illustrates the distribution of Log PFAS Content across different voltage levels, revealing a clear trend where lower voltages (40V–80 V) correspond to higher and more variable PFAS concentrations. As voltage increases beyond 100 V, the data distribution shifts significantly downward, with multiple instances of negative Log PFAS Content, indicating effective PFAS degradation. Supplementary Fig. 2f depicts the average Log PFAS Content as a function of voltage, further supports this observation. The trend shows a monotonic decrease in PFAS content as voltage increases, with a particularly steep decline between 80 V and 100 V, suggesting a critical threshold where PFAS degradation efficiency improves markedly. Beyond 120 V, the downward trend continues but at a slower rate, indicating potentially diminishing returns. These results suggest that higher voltages lead to more effective PFAS reduction, and we hypothesize that the minimum achievable PFAS content will likely occur in a high-voltage range, above 120V. Given this trend, Bayesian optimization should prioritize exploring voltage values beyond 100V, with finer granularity in the range of 120 – 150 V to identify an optimal trade-off between efficiency and diminishing returns.

2. Development of an XGBoost model as a constraint

We trained an XGBoost model as a constraint in the HGBO algorithm. The XGBoost predicts the resistance of the precursor, which is not a measurable parameter. Since the additive materials, additive ratio and mass first determine the resistance before flashing the soil, these three parameters were used as input features to predict the output resistance. In order to select an optimal model, support vector regression [1], Bayesian regression [2], multilayer perceptron [3], random forest [4], and XGBoost regression [5]models were tested using a quintuple cross-validation method. To train

the models, we used a grid search to tune the hyperparameters (Supplementary Table 3), and the performance of the models was evaluated by root mean square error (RMSE) and (R^2). It was observed that data points with resistance values exceeding 10 Ω appeared in only four datasets. Given their limited representation, these data points were considered unsuitable for both expert evaluation and modeling purposes and excluded from the iterative model's training and testing sets (Supplementary Fig. 4).

3. Details of factors in the sigmoid function

Factor P convert a discrete expert confidence score into a smooth, bounded modulation of the uncertainty weight (Supplementary Fig. 3). (1) Definition of the inflection point in the sigmoid function (Eq. 4). We set it as 1.5. This value sets the inflection point of the sigmoid, i.e., $P = 0.5$ when $C_t = 1.5$. With the discrete score set $C_{t,j} \in \{0,1,2,3\}$, it places the transition between 1 and 2, which are intentionally defined as the two intermediate decision states in our scoring scheme. This design makes $C_t \leq 1$ map to $P < 0.5$ (low-to-moderate influence) and $C_t \leq 2$ map to $P > 0.5$ (moderate-to-high influence), while reserving the extremes $C_t = 0$ and 3 for near-saturation. If the value were smaller (e.g., 1.0), then $C_t = 1$ would already lie at the inflection point and $C_t = 2, 3$ would saturate too early, reducing the effective distinction among higher confidence states. If the value were larger (e.g., 2.0), the opposite would occur: $C_t = 2$ would be treated as borderline and $C_t = 0, 1$ would collapse toward the low end. Thus, the parameter of 1.5 aligns the Sigmoid transition point with the intended conceptual boundary between intermediate scores.

(2) Definition of the slope/steepness in the sigmoid function (Eq. 4). We set it as a constant value of 6 to control the steepness of the logistic mapping, i.e., how strongly adjacent discrete confidence levels are separated in P. With the value of 6, the mapping yields a clear but not binary separation: $C_t = 0$ maps close to 0, $C_t = 3$ maps close to 1, and $C_t = 1, 2$ fall on opposite sides of the inflection point with substantial separation (Supplementary Figure 7). If the slope were smaller (e.g., 2–3), the curve would be too shallow and the mapped values for $C_t = 1, 2$ would become insufficiently separated, making the confidence modulation weaker and reducing the practical impact of distinguishing intermediate cases. Conversely, if the slope were larger (e.g., ≥10), the curve would become nearly a step function at 1.5, effectively collapsing the scheme into a hard threshold and eliminating graded behavior between intermediate and extreme confidence states; in preliminary trials this produced overly abrupt changes in candidate ranking. We therefore chose 6 as a moderate slope that provides strong discrimination across $C_{t,j} \in \{0,1,2,3\}$ while preserving a smooth transition rather than a near binary categorization.

4. Details of the multi-branch neural network model

The multi-branch neural network (MBNN) was designed to integrate heterogeneous information sources relevant to PFAS removal efficiency: experimental process conditions, chemical features, and atom-resolved molecular graph structures. The model consists of three distinct branches, each tailored to one data modality, followed by a fusion module and a regression head.

For the **experimental branch**, the stream processes process-related variables, including voltage, resistance and mass. These variables are first standardized using z-score normalization. The input

is passed through three fully connected (FC) layers with dimensions $9 \rightarrow 64 \rightarrow 32 \rightarrow 16$, each followed by ReLU activation and dropout (p = 0.2).

The **chemical features branch** of the model incorporates ten molecular-level features calculated using RDKit, selected to capture key aspects of PFAS structure and reactivity. These include MolWt, the exact molecular weight computed from atomic masses; HeavyAtomCount, the number of non-hydrogen atoms; NumHeteroatoms, which counts atoms other than carbon and hydrogen (e.g., O, N, F, S) and serves as a proxy for polarity; and NumRotatableBonds, the number of single bonds that are not part of rings or terminal groups, reflecting molecular flexibility. LabuteASA refers to the Labute approximate surface area, a surface-based descriptor related to solubility and hydrophobicity. MaxPartialCharge denotes the largest Gasteiger partial atomic charge across all atoms in the molecule, indicative of localized electronic reactivity. MolWt_per_atom is the molecular weight normalized by total atom count (including hydrogens), providing a mass-density-like feature. In addition, PFAS-specific structural characteristics are encoded via C_F_bonds (number of carbon–fluorine bonds), Carboxylic_count (number of –COOH groups), and Sulfonic_count (number of $–SO_3H$ or $–SO_3^-$ groups), with the latter two extracted using SMARTS pattern matching. Together, these features provide interpretable, chemistry-informed representations of PFAS molecules relevant to their thermal degradation behavior. This stream uses the same architecture as the experimental branch: FC $10 \rightarrow 64 \rightarrow 32 \rightarrow 16$ with ReLU and dropout (p = 0.2) after each hidden layer.

In the **molecular graph branch**, which are constructed from the canonical SMILES using RDKit[6] with atoms represented by their atomic numbers and bonds encoded as undirected edges

(bidirectional). For molecules with no bonds, self-loops are added to ensure each atom has at least one connection. Node features (atomic numbers) are embedded into a 64-dimensional space via a linear transformation. Two graph attention convolutional layers[7] are then applied: GATConv1: 64 → 8 × 4 heads = 32 output features, followed by ELU activation and dropout (p = 0.2); GATConv2: 32 → 16 (single head), also with ELU activation and dropout. The node embeddings are aggregated into a single graph-level representation via global mean pooling.

In the **fusion and output layers**, the three 16-dimensional outputs from the respective branches are concatenated into a 48-dimensional vector. This is passed through a fusion module (FC 48 → 32 → 16) and a final prediction head (FC 16 → 8 → 1) with ReLU and dropout layers in between. The output is a scalar prediction of PFAS removal efficiency.

For **training details**, the network is implemented in PyTorch (v2.3) and trained using the Adam optimizer[4] (initial learning rate = 1×10^{-3} weight decay = 1×10^{-5}). Models were trained with a batch size of 64 for a maximum of 500 epochs. Early stopping was applied if the validation loss did not improve for 50 consecutive epochs. All models were trained and evaluated using a scaffold-based train/validation/test split (80/10/10) to ensure structural dissimilarity between training and test molecules. Model performance was evaluated using RMSE and R^2 on the held-out test set.

Regarding **Atom-level importance analysis**, we implemented a perturbation-based saliency method inspired by GNNExplainer[8] to identify atom-level contributions to model predictions. For a given molecule, each atom's embedding was individually zeroed while keeping others fixed. The change in the pooled graph embedding was measured via L2 norm. This score was used to quantify the atom's importance, normalized across all atoms, and visualized on 2D molecular structures

using RDKit's MolDraw2D. Tabulated importance scores along with atomic properties (atomic number, formal charge, degree, hybridization) were exported to Excel files for downstream analysis.

Supplementary Figures

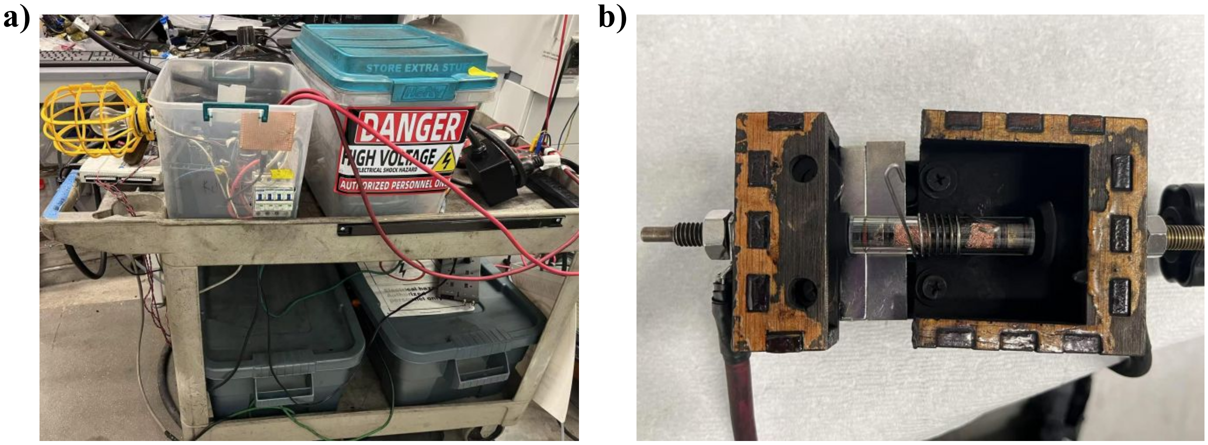

Supplementary Fig. 1. (a) Flash Joule heating (FJH) setup picture. **(b)** Picture of the FJH reaction fixture.

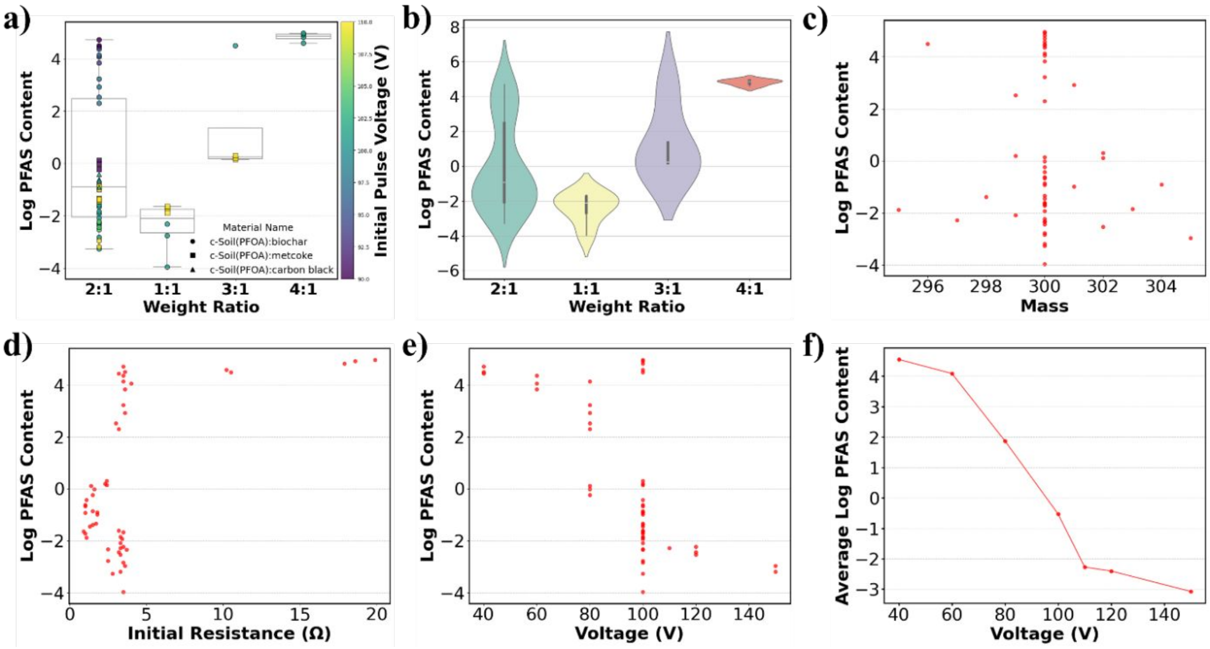

Supplementary Fig. 2. Analysis of initial PFAS flash-treatment data. (**a**) Boxplots of log-transformed PFAS residual concentration for different additive materials: weight ratios (2:1, 1:1, 3:1, 4:1), with individual data points colored by voltage (V). (**b**) Violin plots showing the

distribution of log PFAS residuals across the same weight ratios, with median and interquartile range indicated. (**c**) Scatter plot of log PFAS residual concentration versus sample mass (mg). (**d**) Scatter plot of log PFAS residual concentration versus resistance (Ω). (**e**) Scatter plot of log PFAS residual concentration versus voltage (V). (**f**) Line plot connecting the mean log PFAS residual concentration at each voltage, highlighting the overall trend in treatment efficacy.

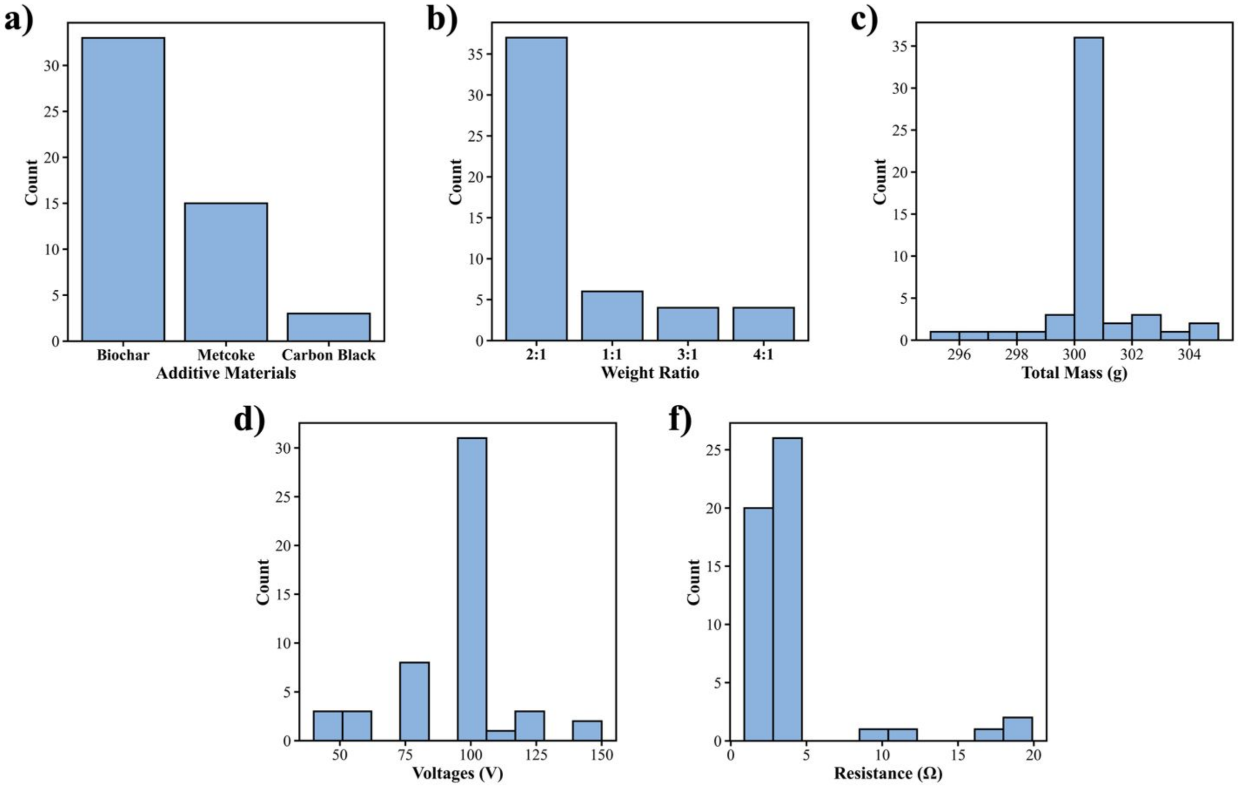

Supplementary Fig. 3. Historical data Distributions of (a) Additives Materials, (b)Weight Ratio, (c)Total Mass (g), (d) Voltages (V), (f) Resistance (Ω).

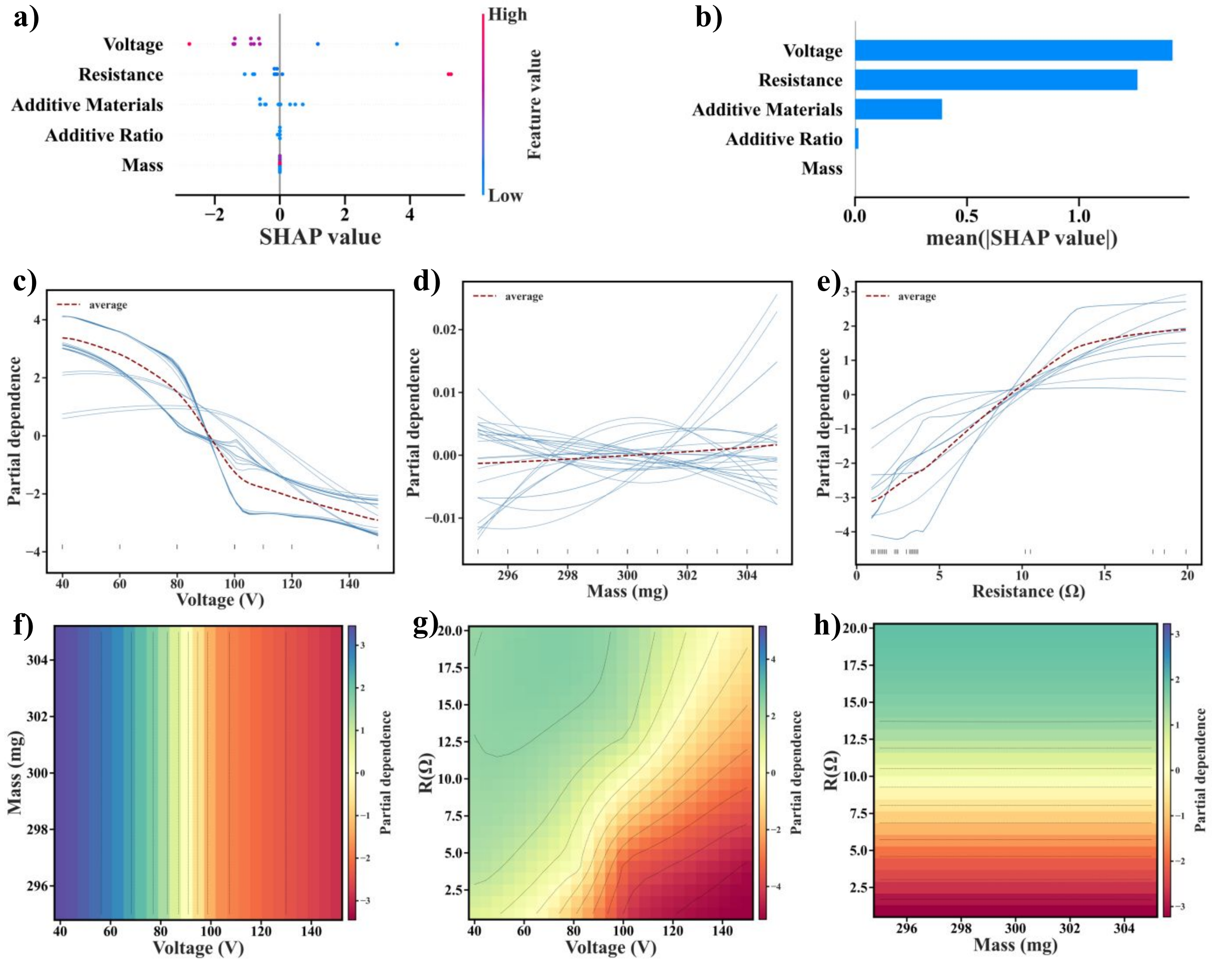

Supplementary Fig. 4. Feature importance and interaction analysis of GP model based on initial data. **(a, b)** SHapley Additive exPlanations values (SHAP) quantifying feature influence on model predictions: (a) SHAP value distribution for each feature, colored by feature value; (b) Average absolute SHAP values, ranking features by relative importance. **(c–e)** Partial dependence plots (PDP) illustrating the isolated effect of individual key variables on predicted PFAS degradation performance:(c) Voltage; (d) Mass; (e) Resistance (Ω). **(f–h)** Two-dimensional PDP contour maps visualizing interaction effects between pairs of significant variables: (f) Mass vs. Voltage; (g) Resistance vs. Voltage; (h) Resistance vs. Mass.

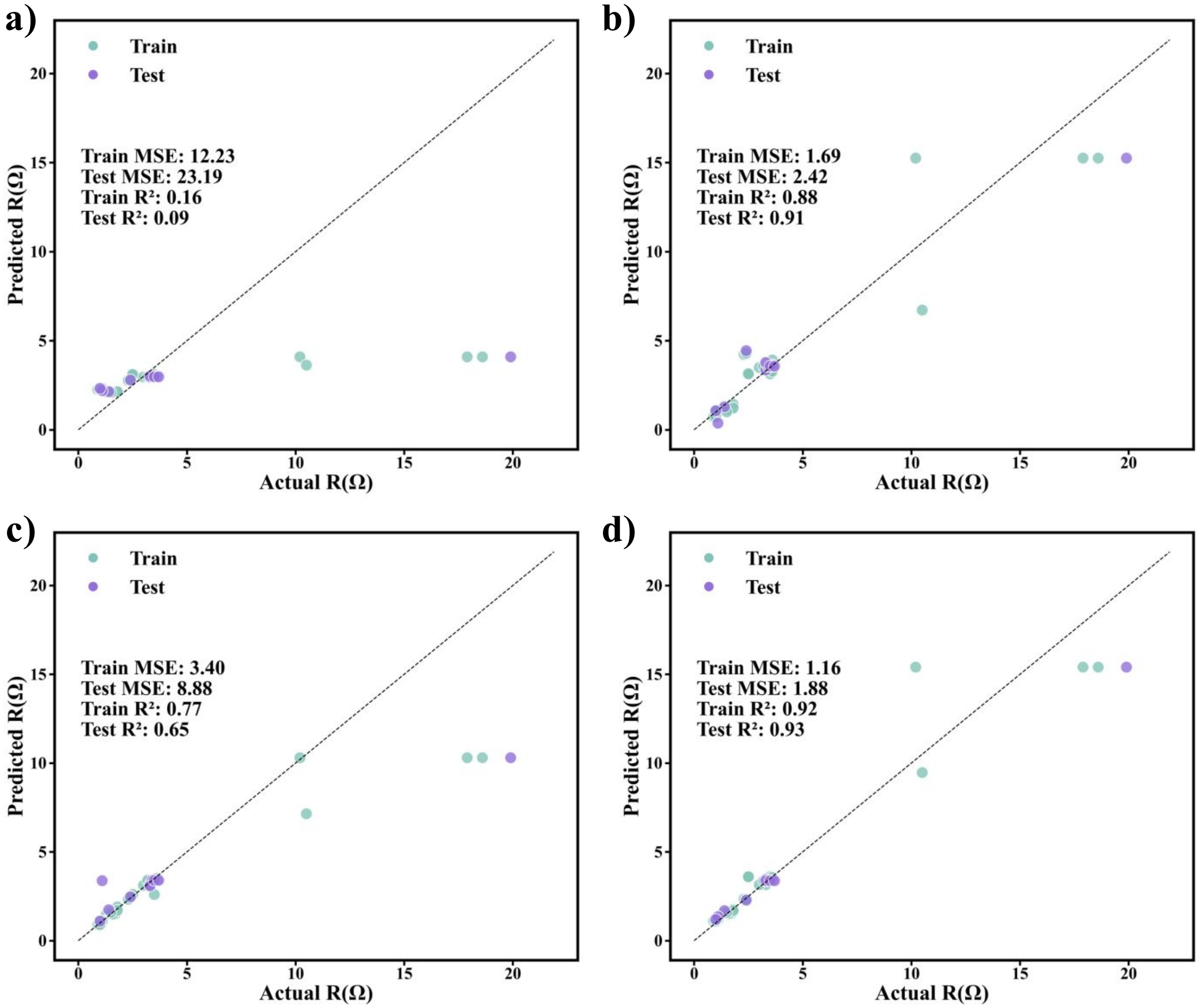

Supplementary Fig. 5. Sample performance of machine learning models: (**a**) Multilayer Perceptron, (**b**) Bayesian Regression, (**c**) Support Vector, (**d**) Random Forest.

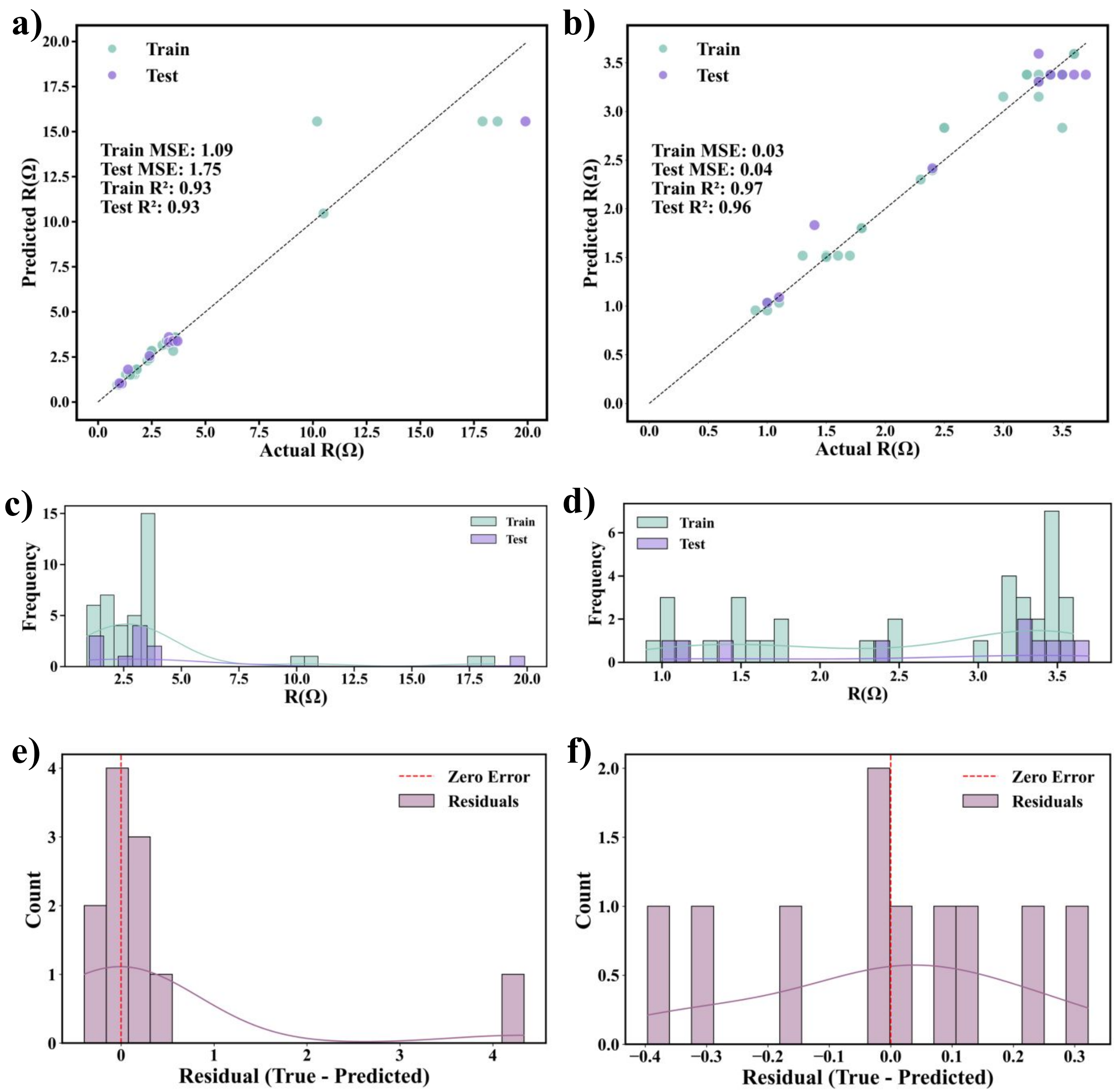

Supplementary Fig. 6. Comparison of XG-R model performance and data distribution before and after data processing. (**a, c, e**) Results on the full dataset (no removal of samples with resistance > 5 Ω): (**a**) Parity plot of predicted versus actual resistance (Ω) for training (green) and test (purple) sets; dashed line denotes perfect agreement (y=x); metrics: train MSE = 1.09, test MSE = 1.75, train R^2=0.93, test R^2=0.93. (**c**) Frequency histogram and kernel density estimate of resistance values in the training and test sets. (**e**) Distribution of residuals (true – predicted) with

zero-error reference line (red dashed) and overlaid density curve. (**b, d, f**) Results after removal of all samples with resistance > 5 Ω: (**b**) Parity plot of predicted versus actual resistance (Ω); metrics: train MSE = 0.03, test MSE = 0.04, train R^2=0.97, test R^2=0.96. (**d**) Frequency histogram and kernel density estimate of the filtered resistance values in the training and test sets. (**f**) Distribution of residuals for the filtered dataset, with zero-error reference line (red dashed) and overlaid density curve.

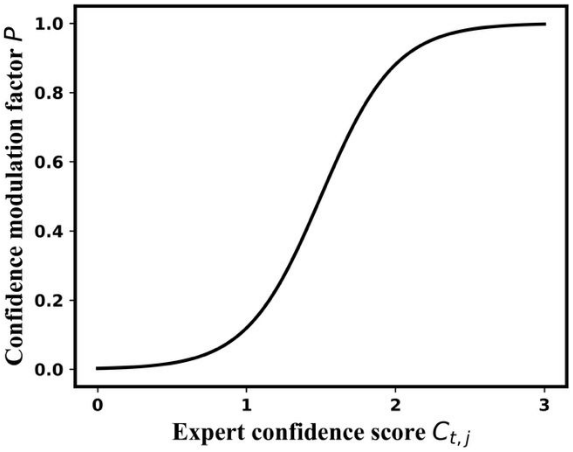

Supplementary Fig. 7. Sigmoid mapping from discrete expert confidence scores to continuous confidence modulation factors.

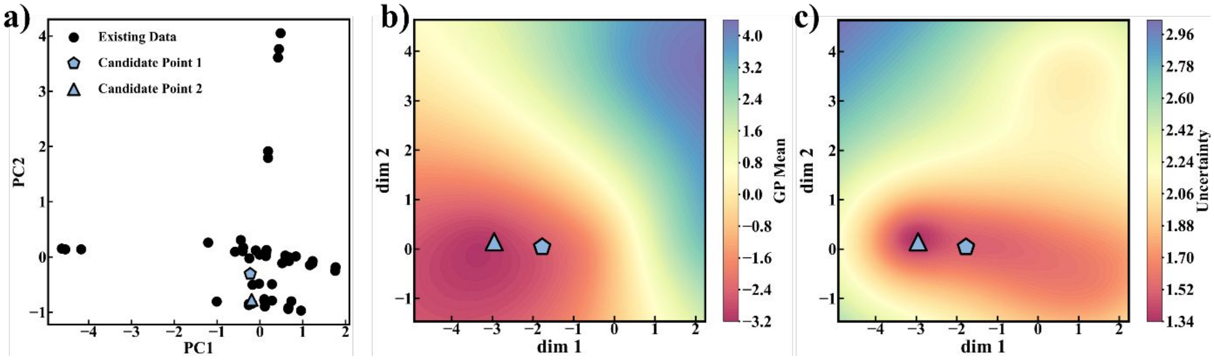

Supplementary Fig. 8. Assignment of confidence scores 1 and 2. (**a**) PCA projection of the input space shows that Candidates 1 and 2 lie near the existing dataset. (**b**) GP-predicted mean values in the reduced 2D space suggest that both candidates yield low predicted performance. (**c**)

GP-predicted uncertainty reveals that Candidate 2 is in a region of significantly higher epistemic uncertainty than Candidate 1. Consequently, Candidate 1 is assigned a confidence score $C_t = 1$, while Candidate 2 is $C_t = 2$ assigned.

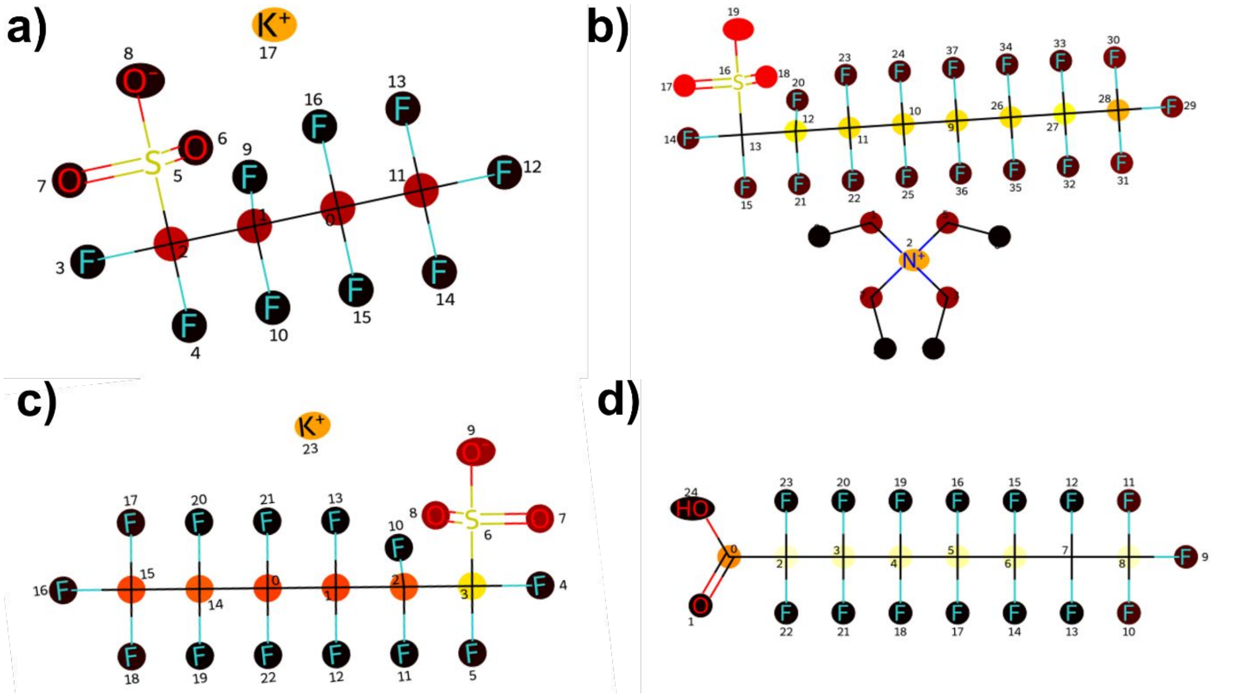

Supplementary Fig. 9. The atomic index and associated element type for each atom in the four PFAS molecules. Atoms that are not explicitly labeled correspond to the carbon chain backbone of the respective molecules: (**a**) PFBS, (**b**) PFOS, (**c**) PFHxS, (**d**) PFOA.

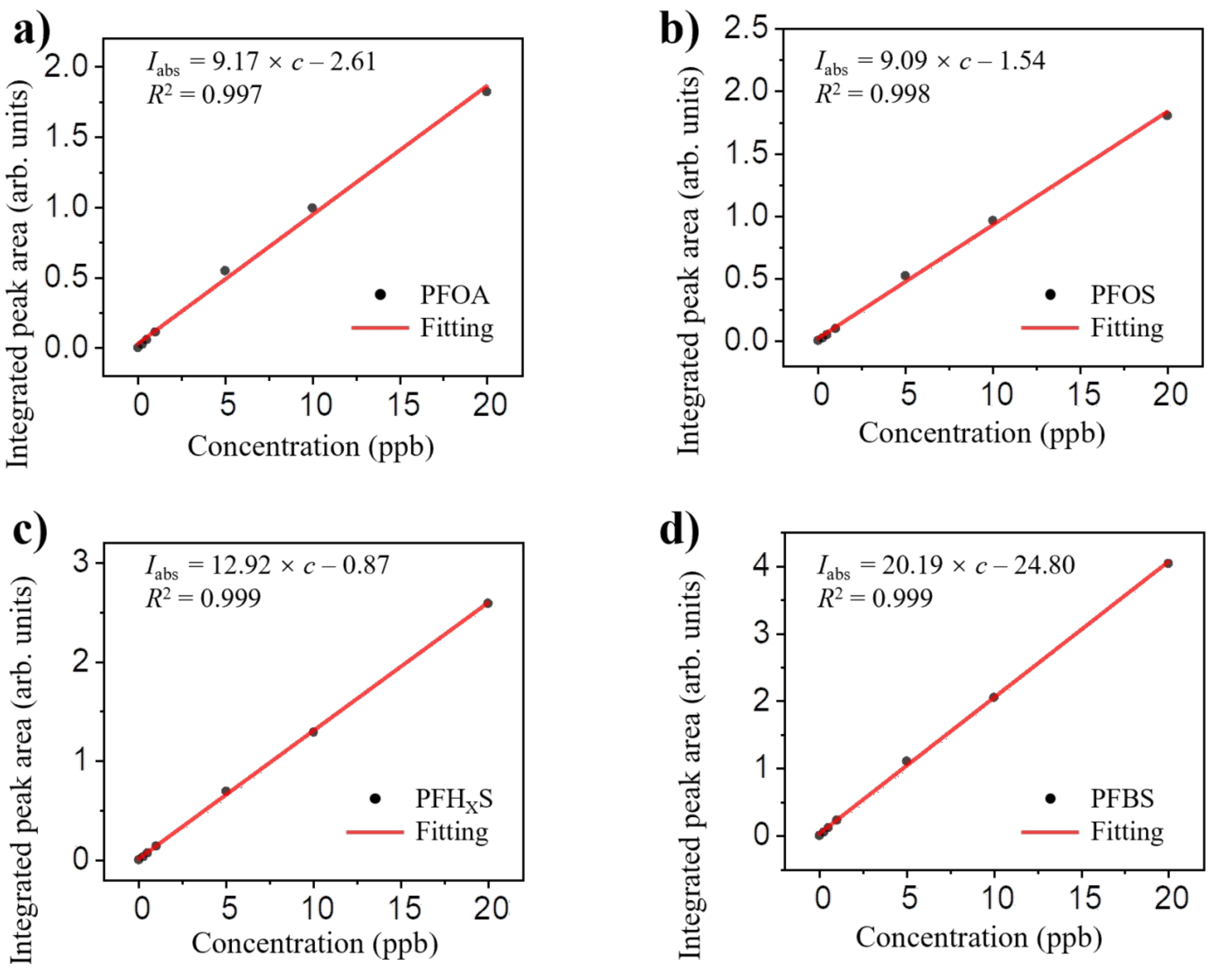

Supplementary Fig. 10. QQQ LC-MS Measurement of PFAS concentration. Calibration curve for fluorine ions by IC. The linearity of the fitting is good ($R^2 > 0.99$), demonstrating the validity of the method for concentration determination. (a) PFOA, (b) PFOS, (c) PFHxS, (d) PFBS.

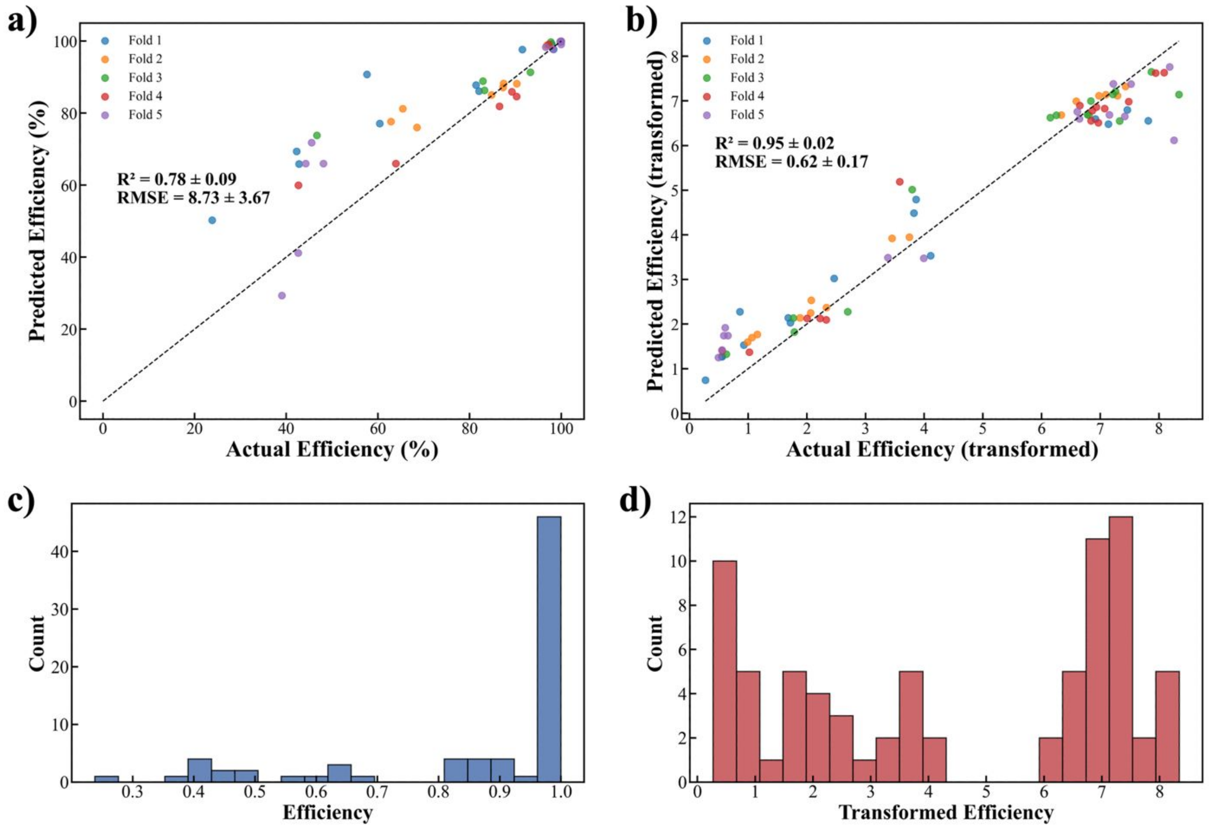

Supplementary Fig. 11. Cross-validation results of the MBNN surrogate under original and transformed efficiency representations. (a–b) 5-fold CV parity plots comparing predicted and actual efficiencies in the original (a) and transformed (b) target spaces. (c–d) Corresponding distributions of efficiency values before (c) and after (d) transformation.

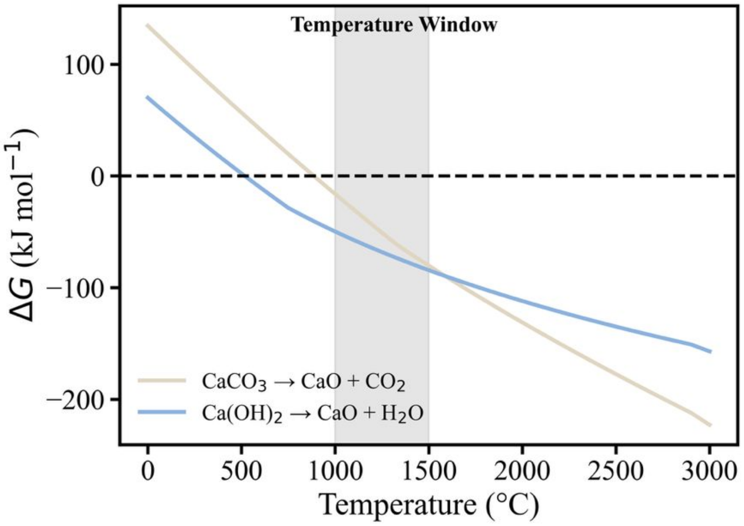

Supplementary Fig. 12. Thermodynamic driving force for CaO formation under FJH-relevant temperatures.

Supplementary Tables

Supplementary Table 1. Details of precursors used for HGBO and DBNN training.

	**HGBO**	**DBNN**
**PFAS Types**	PFOA	PFOA, PFOS, PFHxS, PFBS
**Additive Materials**	Biochar, Metcoke, Carbon	Biochar, Metcoke, Carbon
**Additive Ratio ***	1:1, 2:1, 3:1, 4:1	1:1, 2:1, 3:1, 4:1
**Total Mass**	270 ~ 300 mg	270 ~ 300 mg

* Additive ratio is the weight ratio of PFAS to additive, for example PFOA:Biochar =1:1.

Supplementary Table 2. Molecular properties and fluorine mass ratios of selected PFAS precursors.

**Precursors**	**Formula**	**Molecular weight (g mol^{-1})**	**F mass ratio (%)**
PFOA	$C_8HF_{15}O_2$	414.1	68.8
PFOS	$C_{16}H_{20}F_{17}NO_3S$	629.4	51.3
PFH_xS	$C_6F_{13}O_3SK$	438.2	56.4
PFBS	$C_4F_9O_3SK$	338.2	50.6

Supplementary Table 3. Hyperparameters of regression models in predicting electrical resistance.

**Model**	**Hyperparameters**	**Range**
Multilayer Perceptron (MLP)	activation = 'relu', alpha = 0.01, batch_size = 8, early_stopping = True, hidden_layer_sizes = (200,), learning_rate_init = 0.001, solver = 'adam'	activation = ['relu', 'tanh'], alpha = [0.01, 0.1, 1.0], batch_size = [8, 16, 32], early_stopping = True, hidden_layer_sizes = [(100,), (200,), (100, 50), (200, 100), (300, 150, 50)], learning_rate_init = [0.0001, 0.001], solver = 'adam'
Bayesian Regressor (BR-R)	alpha_1 = 1e-5, alpha_2 = 1e-5, lambda_1 = 1e-7, lambda_2 = 1e-5	alpha_1 = [1e-7, 1e-6, 1e-5], alpha_2 = [1e-7, 1e-6, 1e-5], lambda_1 = [1e-7, 1e-6, 1e-5], lambda_2 = [1e-7, 1e-6, 1e-5]
Support Vector Regressor (SV-R)	C = 20, gamma = 'auto', kernel = 'rbf'	C = [0.1, 1, 10, 20], kernel = ['linear', 'rbf'], gamma = ['scale', 'auto']
Random Forest Regressor (RF-R)	max_depth = None, min_samples_leaf = 1, min_samples_split = 2, n_estimators = 300	n_estimators = [50, 100, 200, 300], max_depth = [None, 10, 15, 20], min_samples_split =[2, 5], min_samples_leaf =[1, 2]
XGBoost Regressor	max_depth = 6, learning_rate = 0.2, n_estimators = 50, subsample = 1.0 colsample_bytree = 0.8,	max_depth = [3, 6, 9], learning_rate = [0.01, 0.1, 0.2], n_estimators = [50, 100, 200], subsample = [0.8, 1.0], colsample_bytree = [0.8, 1.0]

Supplementary Table 4. Candidate experimental conditions recommended by vanilla BO under different uncertainty weights (β).

$\boldsymbol{\beta}$	**Additive Material**	**Weight Ratio**	**Mass**	**Resistance**	**Voltage**
0.1	Biochar	2:1	300	3.5	100
0.25	Biochar	1:1	302	2.6	100
0.5	Biochar	1:1	299	0.8	150
1	Metcoke	1:1	299	0.91	150
2.5	Metcoke	1:1	346	0.91	150
5	Carbon	1:1	348	0.94	150
10	Carbon	4:1	280	2.4	150

Only the first two candidate conditions were experimentally evaluated.

Supplementary Table 5. Detailed acquisition function evaluation and expert confidence scores used in HGBO (n = 3, $\beta = 0.1$**).**

Iteration	Candidates	$\boldsymbol{\mu}$	$\boldsymbol{\sigma}$	$\boldsymbol{C_{t,j}}$	$\boldsymbol{P_{t,j}}$	$\boldsymbol{\alpha}$
1	1	-1.91	1.6	3	3	-2.38
1	2	-1.42	2.2	0	9	-3.38
2	1	-2.67	2.11	3	3	-3.30
2	2	-2.43	2.30	1	9	-4.64

2	3	-2.35	2.45	0	9.5	-4.67

Supplementary Table 6. Input experimental parameters recommended by the vanilla BO.

Iteration	Additive Material	Weight Ratio	Mass	Resistance	Voltage
1	Biochar	2:1	300	3.5	100
1	Biochar	1:1	302	2.6	100
2	Biochar	1:1	302	2.5	100
2	Biochar	2:1	301	3.4	150

Supplementary Table 7. Input experimental parameters recommended by HGBO.

Iteration	Additive Material	Weight Ratio	Mass	Resistance	Voltage
1	Biochar	1:1	301	2.6	150
1	Biochar	1:1	290	2.2	150
2	Biochar	1:1	285	1.9	150
2	Biochar	1:1	280	2.0	150
2	Biochar	1:1	350	2.4	100

Supplementary Table 8. Details of attention scores for all atoms in PFBS. They were derived based on DBNN.

PFBS											
**Atom Index**	5	2	17	0	1	11	8	6	7	13	14
**Atom Symbol**	S	C	K	C	C	C	O	O	O	F	F
**Normalized Score**	1.00	0.70	0.60	0.50	0.49	0.47	0.22	0.22	0.22	0.05	0.05
**Atom Index**	12	4	3	9	10	15	16				
**Atom Symbol**	F	F	F	F	F	F	F				
**Normalized Score**	0.05	0.04	0.04	0	0	0	0				

Supplementary Table 9. Details of attention scores for all atoms in PFOS. They were derived based on DBNN.

PFOS											
**Atom Index**	16	2	13	28	27	12	9	10	26	11	3
**Atom Symbol**	S	N	C	C	C	C	C	C	C	C	C
**Normalized Score**	1.00	0.39	0.39	0.38	0.38	0.36	0.35	0.35	0.35	0.35	0.11
**Atom Index**	5	7	1	18	17	19	29	30	31	15	14
**Atom Symbol**	C	C	C	O	O	O	F	F	F	F	F
**Normalized Score**	0.11	0.11	0.11	0.10	0.10	0.10	0.09	0.09	0.09	0.07	0.07
**Atom Index**	25	24	37	36	35	34	23	22	32	33	21
**Atom Symbol**	F	F	F	F	F	F	F	F	F	F	F
**Normalized Score**	0.05	0.05	0.05	0.05	0.05	0.05	0.05	0.05	0.05	0.05	0.05
**Atom Index**	20	4	8	6	0						
**Atom Symbol**	F	C	C	C	C						
**Normalized Score**	0.05	0.00	0.00	0.00	0.00						

Supplementary Table 10. Details of attention scores for all atoms in PFHxS. They were derived based on DBNN.

PFHxS											
**Atom Index**	6	3	23	14	2	15	1	0	8	7	9
**Atom Symbol**	S	C	K	C	C	C	C	C	O	O	O
**Normalized Score**	1.00	0.70	0.60	0.50	0.49	0.47	0.45	0.45	0.22	0.22	0.22
**Atom Index**	16	18	17	5	4	11	10	20	19	13	12
**Atom Symbol**	F	F	F	F	F	F	F	F	F	F	F
**Normalized Score**	0.05	0.05	0.05	0.04	0.04	0.00	0.00	0.00	0.00	0.00	0.00
**Atom Index**	21	22									
**Atom Symbol**	F	F									
**Normalized Score**	0.00	0.00									

Supplementary Table 11. Details of attention scores for all atoms in PFOA. They were derived based on DBNN.

PFOA											
**Atom Index**	0	2	8	3	6	5	4	7	9	10	11
**Atom Symbol**	C	C	C	C	C	C	C	C	F	F	F
**Normalized Score**	1.00	0.94	0.93	0.61	0.61	0.59	0.58	0.57	0.10	0.10	0.10
**Atom Index**	22	23	24	1	13	12	14	15	18	19	16
**Atom Symbol**	F	F	O	O	F	F	F	F	F	F	F
**Normalized Score**	0.01	0.01	0.01	0.01	0.00	0.00	0.00	0.00	0.00	0.00	0.00
**Atom Index**	17	20	21								
**Atom Symbol**	F	F	F								
**Normalized Score**	0.00	0.00	0.00								

Supplementary References

S1. Drucker, H., Burges, C. J. C., Kaufman, L., Smola, A. & Vapnik, V. Support Vector Regression Machines. in *Advances in Neural Information Processing Systems* vol. 9 (MIT Press, 1996).

S2. Yamashita, T. *et al.* Crystal structure prediction accelerated by Bayesian optimization. *Phys. Rev. Mater.* **2**, 013803 (2018).

S3. Jaeger, S., Fulle, S. & Turk, S. Mol2vec: Unsupervised Machine Learning Approach with Chemical Intuition. *J. Chem. Inf. Model.* **58**, 27–35 (2018).

S4. Breiman, L. Random Forests. *Machine Learning* **45**, 5–32 (2001).

S5. Chen, T. & Guestrin, C. XGBoost: A Scalable Tree Boosting System. in *Proceedings of the 22nd ACM SIGKDD International Conference on Knowledge Discovery and Data Mining* 785–794 (2016). doi:10.1145/2939672.2939785.

S6. Landrum, G. RDKit: A software suite for cheminformatics, computational chemistry, and predictive modeling.

S7. Veličković, P. *et al.* Graph Attention Networks. Preprint at https://doi.org/10.48550/arXiv.1710.10903 (2018).

S8. Ying, R., Bourgeois, D., You, J., Zitnik, M. & Leskovec, J. GNNExplainer: Generating Explanations for Graph Neural Networks. Preprint at https://doi.org/10.48550/arXiv.1903.03894 (2019).
